# Antagonizing miR-455-3p inhibits chemoresistance and aggressiveness in esophageal squamous cell carcinoma

**DOI:** 10.1186/s12943-017-0669-9

**Published:** 2017-06-21

**Authors:** Aibin Liu, Jinrong Zhu, Geyan Wu, Lixue Cao, Zhanyao Tan, Shuxia Zhang, Lili Jiang, Jueheng Wu, Mengfeng Li, Libing Song, Jun Li

**Affiliations:** 1 Program of Cancer Research, Affiliated Guangzhou Women and Children’s Hospital, Department of Biochemistry, Zhongshan School of Medicine, Sun Yat-sen University, 74 Zhongshan Road II, Guangzhou, Guangdong 510080 China; 2 State Key Laboratory of Oncology in Southern China, Department of Experimental Research, Cancer Center, Sun Yat-sen University, Guangzhou, 510060 China; 3 Key Laboratory of Protein Modification and Degradation, School of Basic Medical Sciences, Affiliated Cancer Hospital & Institute of Guangzhou Medical University, Guangzhou, China; 4 Department of Microbiology, Zhongshan School of Medicine, Sun Yat-sen University, Guangzhou, China

**Keywords:** miR-455-3p, Chemoresistance, Esophageal squamous cell carcinoma, OncomiR

## Abstract

**Background:**

The plasticity of cancer stem cells (CSCs)/tumor-initiating cells (T-ICs) suggests that multiple CSC/T-IC subpopulations exist within a tumor and that multiple oncogenic pathways collaborate to maintain the CSC/T-IC state. Here, we aimed to identify potential therapeutic targets that concomitantly regulate multiple T-IC subpopulations and CSC/T-IC-associated pathways.

**Methods:**

A chemoresistant patient-derived xenograft (PDX) model of human esophageal squamous cell carcinoma (ESCC) was employed to identify microRNAs that contribute to ESCC aggressiveness. The oncogenic effects of microRNA-455-3p (miR-455-3p) on ESCC chemoresistance and tumorigenesis were examined by in vivo and in vitro chemoresistance, tumorsphere formation, side-population, and in vivo limiting dilution assays. The roles of miR-455-3p in activation of the Wnt/β-catenin and transforming growth factor-β (TGF-β)/Smad pathways were determined by luciferase and RNA immunoprecipitation assays.

**Results:**

We found that miR-455-3p played essential roles in ESCC chemoresistance and tumorigenesis. Treatment with a miR-455-3p antagomir dramatically chemosensitized ESCC cells and reduced the subpopulations of CD90^+^ and CD271^+^ T-ICs via deactivation of multiple stemness-associated pathways, including Wnt/β-catenin and TGF-β signaling. Importantly, miR-455-3p exhibited aberrant upregulation in various human cancer types, and was significantly associated with decreased overall survival of cancer patients.

**Conclusions:**

Our results demonstrate that miR-455-3p functions as an oncomiR in ESCC progression and may provide a potential therapeutic target to achieve better clinical outcomes in cancer patients.

**Electronic supplementary material:**

The online version of this article (doi:10.1186/s12943-017-0669-9) contains supplementary material, which is available to authorized users.

## Background

Over the last two decades, accumulating evidence has implicated a minority population of cells within tumors, termed cancer stem cells (CSCs) or tumor-initiating cells (T-ICs), in cancer recurrence, metastasis, and conventional therapy resistance, which are the critical determinants of prognosis in human cancers [1–4]. T-ICs were first identified in acute myeloid leukemia, and have subsequently been found in a variety of human solid tumors, such as breast, brain, colon, prostate, liver, and pancreatic cancers [1, 5–12]. T-IC subpopulations, which reside at the apex of the hierarchy of mixed tumor cells, exhibit enhanced tumor-initiating capabilities, extensive self-renewal, and intrinsic resistance to conventional therapies, properties that contribute to tumor initiation and maintenance, regression, and chemoradiotherapy failure [1, 5–12]. Therefore, the development of T-IC-targeting therapeutics represents a promising strategy to improve clinical outcomes in cancer patients.

Interestingly, the distinct subpopulations of tumor cells isolated using different cell-surface markers demonstrate enhanced tumor-initiating capabilities, suggesting that multiple T-IC subpools exist within a tumor. For instance, Lee et al. reported that CD24^+^ hepatocellular carcinoma (HCC) cells were critical for the initiation, self-renewal, and metastasis of HCC. Likewise, Ma et al. found that CD133^+^ HCC cells display a preferential capacity for self-renewal and in vivo tumor initiation [12, 13]. Therefore, targeting a single T-IC subpopulation within a tumor may be insufficient for effective cancer treatment.

The identification and characterization of T-ICs has revealed that multiple intracellular signaling pathways, activated by heritable genetic and epigenetic alterations and the tumor microenvironment, are involved in the induction and maintenance of T-IC-like traits and are constitutively overactivated in T-ICs [14, 15]. For example, excessive Wnt/β-catenin signaling is reportedly required for the maintenance of T-IC capabilities in colon cancer, cutaneous cancer, glioma, and mixed-lineage leukemia [16–19]. Similarly, overactivated transforming growth factor-β (TGF-β) signaling is essential for the stemness of glioma-initiating cells and maintenance of skin T-ICs and leukemia-initiating cells [1–3]. Given the prominence of stemness-associated pathways in cancer initiation and progression, multiple inhibitors of these signaling cascades have been developed and tested in clinical trials [14]. However, many pathways are simultaneously overactivated in the same T-IC population and actively cooperate to maintain the T-IC state. For instance, the Wnt/β-catenin and TGF-β signaling pathways collaborate to maintain mammary T-ICs [20–23]. Therefore, targeting a single signaling pathway may be insufficient to eradicate T-ICs.

Through their ability to simultaneously repress multiple target genes, microRNAs (miRNAs) play circuital roles in the maintenance of T-IC traits and chemoresistance [24–26], highlighting their potential as anti-cancer agents. Herein, we report that treatment with antagomir-455-3p chemosensitized esophageal squamous cell carcinoma (ESCC) cells and reduced the CD90^+^ and CD271^+^ T-IC subpopulations via the inhibition of multiple stemness-associated pathways, including Wnt/β-catenin and TGF-β signaling. Our findings provide an attractive therapeutic approach for targeting T-ICs to achieve better clinical outcomes in cancer patients.

## Methods

### Cell lines and primary cell culture

Primary ESCC cells were isolated from fresh ESCC tissues, which according to previous report [27]. The ESCC cell lines Eca109 and Kyse30, lung cancer cell line H157 and gastric cancer cell line AGS were grown in DMEM (Invitrogen, Carlsbad, CA) supplemented with 10% fetal bovine serum (HyClone, Logan, UT). All cell lines were authenticated by short tandem repeat (STR) fingerprinting at Medicine Lab of Forensic Medicine Department of Sun Yat-Sen University (China).

### Tissue specimens and patient information

A total of 207 paraffin-embedded, archived ESCC and specimens were clinically and histopathologically diagnosed at the Sun Yat-Sen University Cancer Center from 2000 to 2010. ESCC and adjacent non-tumor tissues were obtained from resected tumors and adjacent non-tumor esophageal tissues, respectively, provided by Sun Yat-Sen University Cancer Center and confirmed by pathological review. Clinical information on the samples is summarized in Additional file 1: Table S1. For the use of these clinical materials for research purposes, prior patient consent and approval from the Institutional Research Ethics Committee were obtained.

### Chemoresistant tumor model

All experimental procedures were approved by the Institutional Animal Care and Use Committee of Sun Yat-sen University. Tumors were initiated by subcutaneous implantation of isolated esophageal tumor cell populations, coated with matrigel and media in a 1:1 ratio, into NOD/Shi-*scid*/IL-2Rγ^null^ **(**NOG**)** mice (5 weeks of age, 18–20 g), which were purchased from the Central Institute for Experimental Animals (Kanagawa, Japan). Treatment was initiated once the size of the xenograft reached approximately 4 mm in diameter. The mice were randomly assigned into two groups, which were intraperitoneally treated with cisplatin (CDDP; 5 mg/kg) or PBS (control) twice per week for 3 weeks, then tumor cells were isolated and re-inoculated subcutaneously into NOG mice for the next round of CDDP or control treatment, until to the fourth round that CDDP treatment did not further reduce the tumor size.

### In vivo tumorigenesis experiments

Different numbers of cells were inoculated with Matrigel (final concentration 25%) subcutaneously into the inguinal folds of NOG mice. Tumor volume was determined using external caliper and calculated using the eq. (L × W^2^)/2. Tumors were examined twice weekly; length, width, and thickness measurements were obtained with calipers and tumor volumes calculated. Tumor volume was calculated using the eq. (L*W^2^)/2. For ESCC cells expressing luciferase, bioluminescent imaging was performed using Xenogen IVIS Spectrum (Caliper Life Sciences).

### Plasmids, virus constructs, mimic and antagomir

The human miR-455-3p gene was PCR-amplified from genomic DNA and cloned into a pMSCV-puro retroviral vector. The miR-455-3p anti-sense was cloned into miRZip plasmid purchased from System Biosciences (San Francisco, CA). The 3′-UTR regions of human dickkopf WNT signaling pathway inhibitor 3 (DKK3), glycogen synthase kinase 3β (GSK3β), SMAD specific E3 ubiquitin protein ligase 2 (Smurf2) and protein phosphatase, Mg2+/Mn2+ dependent 1A (PPM1A), generated by PCR amplification from genomic DNA isolated from HEK293T, were cloned into the pGL3-luciferase reporter plasmid (Promega, Madison, WI). The point mutations in the tentative miR-455-3p-binding seed regions were created using the Strategene QuickChange Mutagenesis Kit (Stratagene, La Jolla, CA). Antagomir-455-3p was purchased from RIBOBIO company (GuangZhou, China). Transfection of siRNAs or plasmids was performed using Lipofectamine 3000 reagent (Invitrogen, Carlsbad, CA) according to the manufacturer’s instructions. Stable cell lines expressing pMSCV-miR-455-3p or miRZip-455-3p was generated by retroviral infection using HEK293T cells, and selected with 0.5 μg/ml puromycin for 10 days.

### RNA immunoprecipitation (RIP) assay

Cells were co-transfected with a plasmid that encodes HA-Ago1 and miR-455-3p (100 nM), followed by HA-Ago1 IP using an anti-HA antibody. Real-time PCR analysis of the immunoprecipitated material was used to test the association of the indicated mRNA with the RISC complex.

### Microarray data process and visualization

Microarray data were downloaded from the The Cancer Genome Atlas (TCGA) datasets (http://cancergenome.nih.gov/) and GEO data base (http://www.ncbi.nlm.nih.gov/geo/) using indicated accession numbers. Microarray data described herein have been deposited in the National Center for Biotechnology Information Gene Expression Omnibus with accession number GSE83362. Microarray data extract were performed on MeV 4.6 (http://www.tm4.org/mev/). Gene set enrichment analysis (GSEA) was performed on GSEA 2.0.9 (http://www.broadinstitute.org/gsea/).

### Statistical analysis

Statistical tests for data analysis included Fisher’s exact test, log-rank test, Chi-square test, and Student’s 2-tailed t test. Multivariate statistical analysis was performed using a Cox regression model. Statistical analyses were performed using the SPSS 21.0 statistical software package. Data represent mean ± SD. *P* values of 0.05 or less were considered statistically significant.

## Results

### Chemoresistant ESCC cells possess T-IC-like traits

**Fig. 1:**
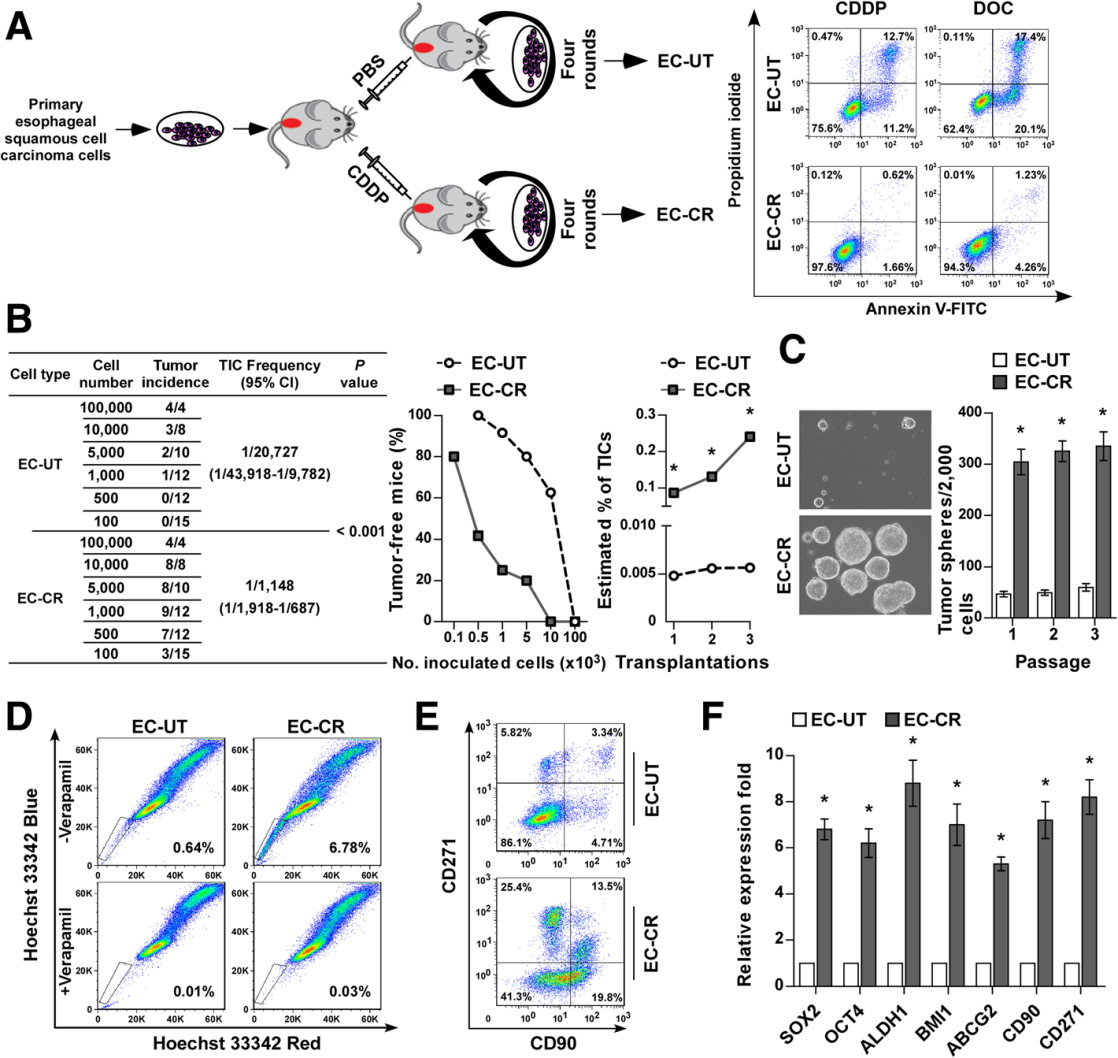
Chemoresistant ESCC possess T-ICs-like traits. **a** *Left panel*: Schematic representation of the establishment of a patient-derived xenograft mouse model to enrich clinical ESCC T-ICs. NOG mice were orthotopically implanted with clinical human ESCC cells and treated with PBS (control) or CDDP (5 mg/kg, twice a week) for 3 weeks. Tumor cells were then re-inoculated subcutaneously into NOG mice for the next round of treatment until CDDP treatment no longer reduced tumor size (fourth round of treatment). *Right panel*: Annexin V-FITC/PI staining of CDDP-resistant ESCC (EC-CR) and CDDP-untreated ESCC (EC-UT) cells treated with CDDP (20 μM) or DOC (1.5 nM) for 24 h. **b** *Left table* and *middle graph*: The frequency of T-ICs. *Right graph*: The estimated percentage of T-ICs among EC-CR and EC-UT cells during serial transplantations. **c** Representative images (*left*) and quantification (*right*) of tumorspheres formed by the indicated cells. **d**, **e** Flow cytometry analysis of the percentage of side-population cells (**d**) and CD90^+^ and CD271^+^ subpopulations (**e**) of the indicated cells. **f** Real-time PCR analysis of the mRNA expression of the indicated transcripts in EC-CR and EC-UT cells. Each bar represents the mean ± SD of three independent experiments. * *P* < 0.05

To enrich the proportion of T-ICs in clinical ESCC tissues, a chemoresistant model of human ESCC tissue in immunodeficient NOD/Shi-*scid*/IL-2Rγ^null^ **(**NOG**)** mice was established, as previously reported [28–30]. As shown in the schematic in Fig. 1a (left), NOG mice bearing subcutaneous tumor xenografts derived from clinical ESCC cells were intraperitoneally treated with Cisplatin (CDDP) or phosphate-buffered saline (PBS) twice weekly for 3 weeks. Tumor cells were then isolated and re-inoculated subcutaneously into NOG mice for the next round of treatment. In the fourth round of treatment, the volume of the tumors in both the CDDP- and PBS-treated groups was approximately the same, suggesting that the cells in the CDDP-treated tumors were becoming resistant to CDDP. As expected, the CDDP-resistant ESCC (EC-CR) cells isolated from CDDP-treated tumors displayed much higher resistance to the chemotherapeutic drugs CDDP and docetaxel (DOC) than the CDDP-untreated ESCC (EC-UT) cells isolated from PBS-treated tumors (Fig. 1a, right). These results indicate the successful establishment of chemoresistant ESCC cells.

Furthermore, in vivo limiting dilution assay showed EC-CR cells exhibited an increased capacity to form tumors compared with EC-UT cells. This difference further increased during serial transplantation (Fig. 1b), indicating that the chemoresistant ESCC cells possessed enhanced tumor-forming and self-renewal abilities. Consistently, in an in vitro tumorsphere formation assay, EC-CR cells formed significantly larger and more numerous tumorspheres than EC-UT cells (Fig. 1c). The proportions of CD90^+^ and CD271^+^ cells, previously identified as T-IC subpopulations in ESCC [31, 32], and side-population (SP) cells increased among EC-CR cells compared with EC-UT cells (Fig. 1d and e). Moreover, the expression of stemness-associated genes, such as *SOX2*, *OCT4*, *ABCG2*, and *BMI1*, was significantly elevated in EC-CR cells compared with EC-UT cells (Fig. 1f). These results demonstrate that EC-CR cells possess T-IC-like traits.

### miR-455-3p promotes chemoresistance and tumorigenesis of ESCC cells

**Fig. 2:**
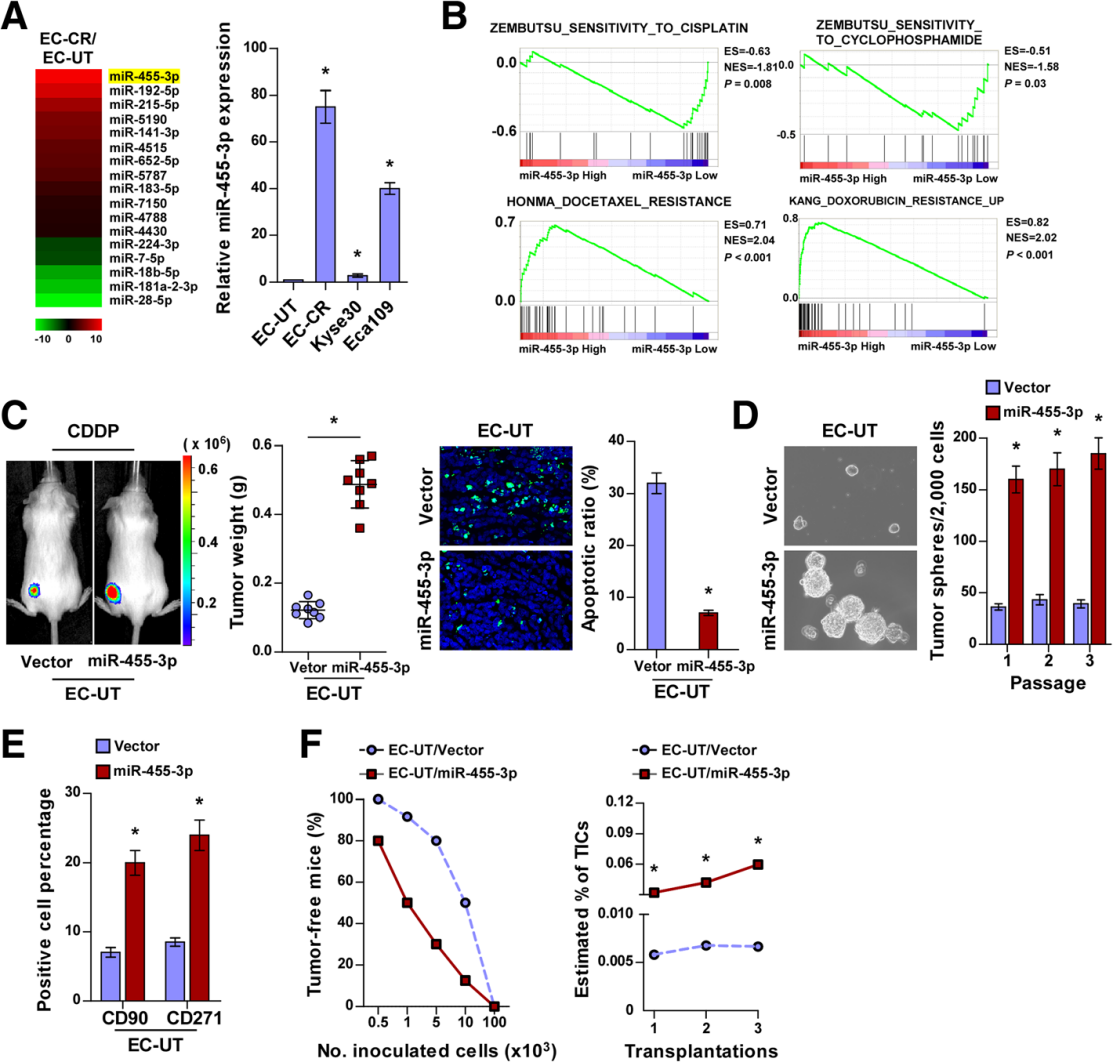
miR-455-3p enhances ESCC chemoresistance and tumorigenesis. **a** miRNA array (*left*) and real-time PCR (*right*) analyses of miR-433-5p in the indicated cells. Microarray data accession number is GSE83362. Transcript levels were normalized to *U6* expression. **b** GSEA of TCGA datasets indicating that miR-455-3p expression was significantly correlated with chemoresistance gene signatures. **c** Representative images of tumor-bearing mice (*left*), weight of xenografts (*middle*), and quantification of TUNEL-stained cells (*right*) in the indicated tumors. **d** Representative images (*left*) and quantification (*right*) of tumorspheres formed by the indicated cells. **e** Flow cytometry analysis of the percentages of CD90^+^/CD271^+^ subpopulations of the indicated cells. **f** The frequency (*left*) and estimated percentage (*right*) of T-ICs among the indicated cells during serial transplantations. Each bar represents the mean ± SD of three independent experiments. * *P* < 0.05

Recently, miRNAs have demonstrated potential as novel therapeutic targets for cancer treatment [24, 26, 33]. We conducted miRNA profiling in EC-CR and EC-UT cells (GSE83362) and showed that miR-455-3p expression was significantly higher in EC-CR cells than in EC-UT cells (Fig. 2a). Strikingly, gene set enrichment analysis (GSEA) of The Cancer Genome Atlas (TCGA) datasets revealed that ESCC exhibiting high miR-455-3p expression was enriched in resistance gene sets for chemotherapeutic drugs such as CDDP, DOC, doxorubicin, gefitinib, dasatinib, cyclophosphamide, and vincristine, whereas ESCC exhibiting low miR-455-3p expression was enriched in chemotherapy sensitive gene sets (Fig. 2b and Additional file 2: Figure S1A), suggesting that miR-455-3p contributes to ESCC chemoresistance. As predicted, overexpressing miR-455-3p conferred resistance to CDDP and DOC in EC-UT and Kyse30 cells, but silencing miR-455-3p enhanced the sensitivity of EC-CR and Eca109 cells to chemotherapeutic agents (Additional file 2: Figure S1B). Moreover, in an in vivo chemoresistance assay, the tumors formed by miR-455-3p-overexpressing cells upon CDDP treatment were larger and contained fewer apoptotic cells than the control tumors (Fig. 2c and Additional file 2: Figure S1C). Collectively, these results suggest that miR-455-3p plays an important role in ESCC chemoresistance.

Consistent with the GSEA of TCGA datasets, through which miR-455-3p expression was found to be significantly associated with stemness signatures, miR-455-3p-transduced cells formed larger and more numerous tumorspheres containing higher proportions of CD90^+^/CD271^+^ and SP cells than control cells (Fig. 2d and e and Additional file 2: Figure S1E and G). Importantly, limiting dilution and serial transplantation assays revealed a significantly higher tumor incidence and greater tumor-forming capacity in miR-455-3p-transduced ESCC cells than in control cells (Fig. 2f), indicating that miR-455-3p contributes to the tumor-forming and self-renewal capabilities of ESCC cells.

### Silencing miRNA-455-3p chemosensitizes ESCC cells and reduces T-ICs-like traits

**Fig. 3:**
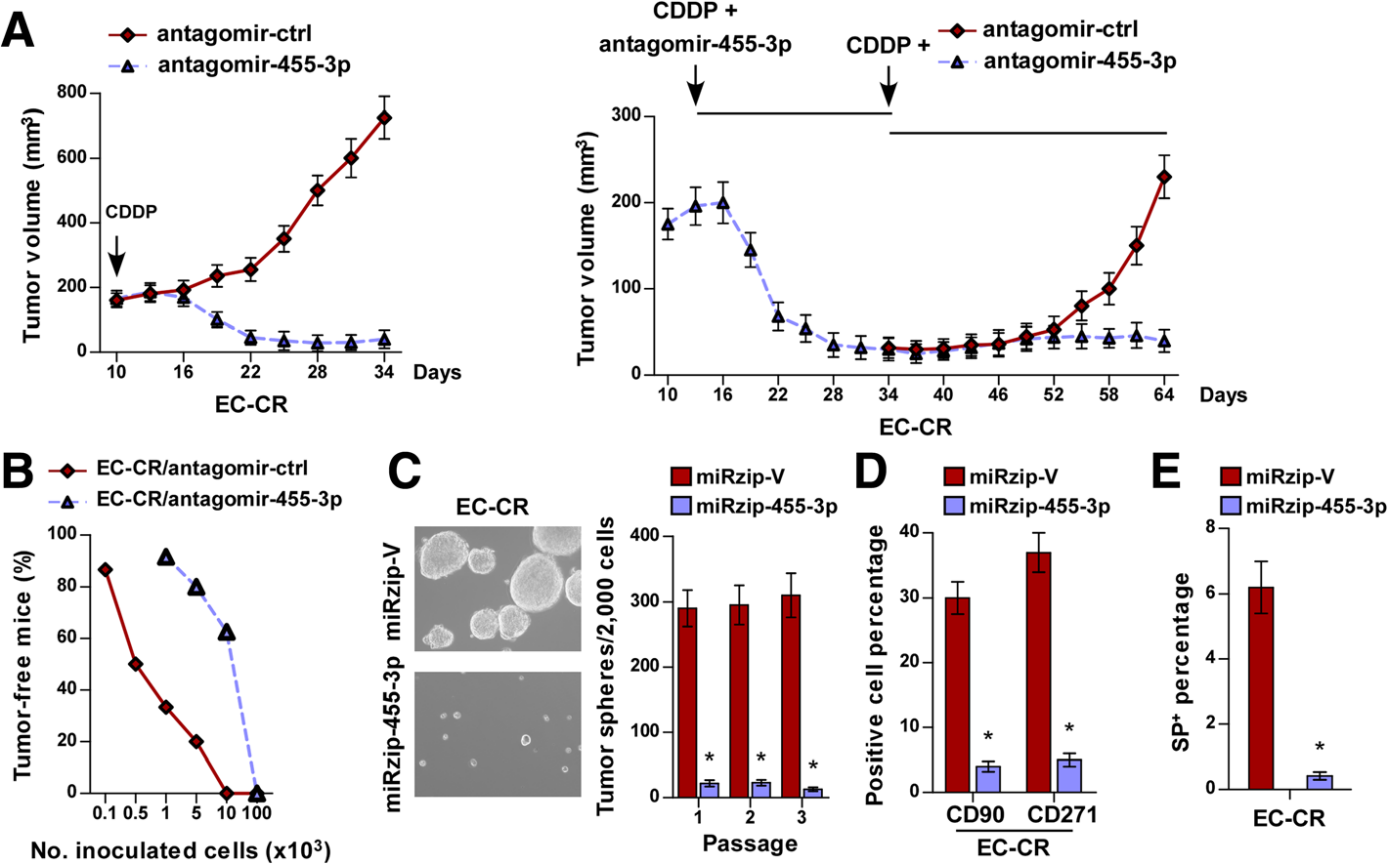
Inhibition of miR-455-3p chemosensitizes ESCC cells and reduces stem cell-like traits. **a** Representative tumor growth curves of xenografts derived from CDDP-resistant ESCC cells co-treated with CDDP (5 mg/kg) and antagomir-control or with CDDP (5 mg/kg) and antagomir-455-3p on the indicated days (*left*). To establish the contribution of miR-455-3p, antagomir-455-3p treatment was stopped on Day 34, but CDDP treatment was continued (*right*). **b** The frequency of T-ICs in the indicated cells. **c** Representative images (*left*) and quantification (*right*) of tumorspheres formed by the indicated cells. **d**, **e** Flow cytometry analysis of the percentages of the CD90^+^ and CD271^+^ subpopulations (**d**) and SP (**e**) of the indicated cells. Each bar represents the mean ± SD of three independent experiments. * *P* < 0.05

As expected, CDDP-treatment alone had no effect on the tumor growth of EC-CR cells in NOG mice, whereas co-treatment with CDDP and antagomir-455-3p had an inhibitory effect on tumor growth (Fig. 3a and b). Strikingly, EC-CR-cell tumor growth recurred after the cessation of treatment with the miR-455-3p antagomir, despite continued CDDP treatment (Fig. 3c). Similarly, silencing of miR-455-3p drastically enhanced the inhibitory effect of CDDP on tumor growth in Eca109 cells and increased the apoptotic rate in Eca109 tumors (Additional file 3: Figure S2A and B). These results demonstrate that silencing miR-455-3p causes the chemosensitization of ESCC cells.

Consistent with the enhancement of T-IC-like traits in ESCC cells by miR-455-3p, the antagomir-455-3p significantly repressed the tumor-initiating and self-renewal abilities of ESCC cells in vivo, and exerted an inhibitory effect on the tumorsphere-forming abilities of EC-CR and Eca109 cells in vitro (Fig. 3b and c and Additional file 3: Figure S2C), providing further evidence that miR-455-3p contributes to the T-IC-like traits of ESCC cells. Concordantly, silencing miR-455-3p in EC-CR and Eca109 cells significantly decreased the proportion of CD90^+^/CD271^+^ and SP cells and reduced the expression of stemness-associated factors (Fig. 3d and e and Additional file 3: Figure S2D and F).

### miRNA-455-3p overexpression correlates with poor prognosis in ESCC patients

**Fig. 4:**
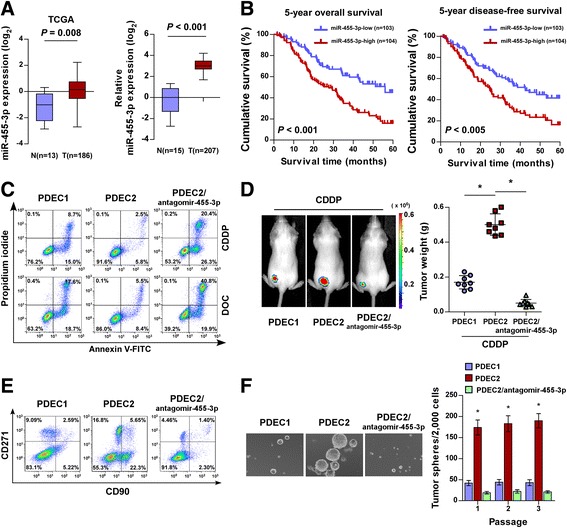
Silencing miR-455-3p chemosensitizes and reduces the tumorigenesis of PDEC cells. **a** Analysis of miR-455-3p expression in TCGA (*n* = 199; *P =* 0.008; left) and in collected clinical samples (*n* = 222; *P <* 0.001; right) indicating that miR-455-3p was significantly upregulated in primary ESCC. **b** Kaplan–Meier analysis of overall and disease-free survival curves for patients with ESCC exhibiting low or high miR-455-3p expression. **c** Annexin V-FITC/PI staining of the indicated cells treated with cisplatin (20 μM) or DOC (1.5 nM) for 24 h. **d** Representative images of tumor-bearing mice (*left*) and weight of xenografts (*right*) derived from the indicated CDDP-treated cells. **e** Flow cytometry analysis of the percentages of CD90^+^/CD271^+^ subpopulations in the indicated cells. **f** Representative images (*left*) and quantification (*right*) of tumorspheres formed by the indicated cells. Each bar represents the mean ± SD of three independent experiments. * *P <* 0.05

Consistent with TCGA analysis, the results of which showed that miR-455-3p was markedly upregulated in ESCC and correlated with shorter overall and disease-free survival in patients with ESCC (Fig. 4a and Additional file 4: Figure S3A and B), real-time polymerase chain reaction (PCR) analysis showed that miR-455-3p was significantly upregulated in 207 ESCC specimens compared with 15 normal esophageal tissues, and that miR-455-3p levels were positively correlated with clinical stage (*P* < 0.001), TNM classification (*P* < 0.001), and histologic differentiation (*P* = 0.002) in patients with ESCC (Fig. 4a and Additional file 1: Table S2). Importantly, patients with higher miR-455-3p expression experienced shorter overall and disease-free survival, whereas patients with lower miR-455-3p expression experienced longer overall and disease-free survival (*P* < 0.05; Fig. 4b). Additionally, miR-455-3p expression was recognized as an independent prognostic factor (*P* < 0.001; Additional file 1: Table S3). Thus, both TCGA analysis and these results suggest a potential link between miR-455-3p overexpression and ESCC progression.

### Silencing microRNA-455-3p chemosensitizes and reduces tumorigenesis of patient-derived esophageal squamous cell carcinoma cells

The contribution of miR-455-3p to chemoresistance and tumorigenesis was further demonstrated in patient-derived esophageal squamous cell carcinoma (PDEC) cells, which more closely resemble the cells present in the tumor masses of cancer patients [34]. As shown in Fig. 4c and d and Additional file 4: Figure S3C, both in vitro and in vivo chemoresistance experiments indicated that PDEC2 cells with higher miR-455-3p expression exhibited greater resistance to chemotherapeutic drugs than PDEC1 cells with lower miR-455-3p expression. Importantly, silencing miR-455-3p dramatically enhanced the sensitivity of PDEC2 cells to chemotherapeutic drugs, and reduced the percentages of CD90^+^/CD271^+^ and SP cells and tumorsphere-forming capability of PDEC2 cells (Fig. 4d and f and Additional file 4: Figure S3D). Together, these results support the contribution of miR-455-3p to the chemoresistance and tumorigenesis of ESCC.

### miRNA-455-3p activates the Wnt/β-catenin and TGF-β/Smad pathways

**Fig. 5:**
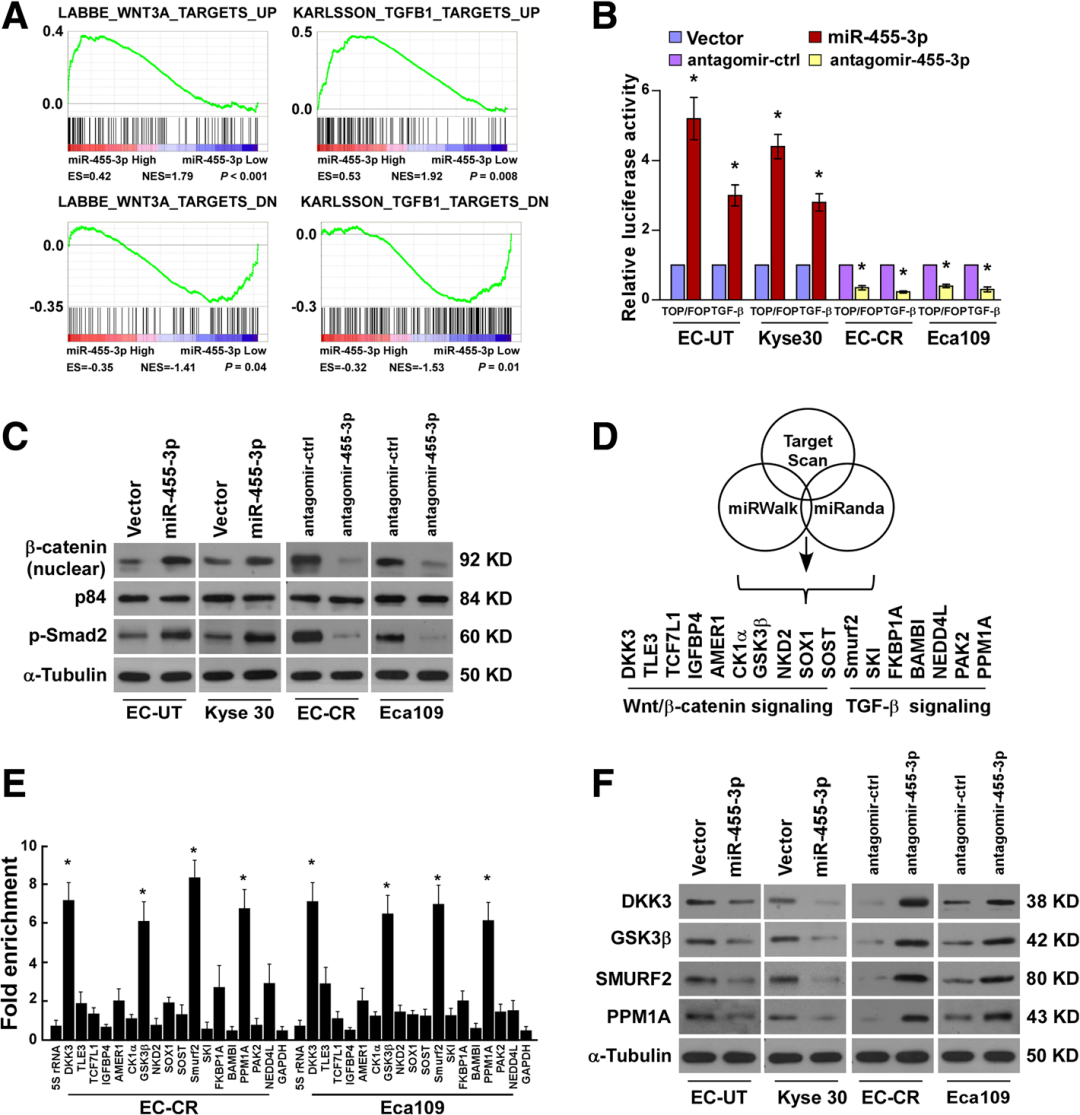
miR-455-3p activates T-IC-associated signaling pathways. **a** GSEA analysis of TCGA indicating that miR-455-3p expression is significantly correlated with gene signatures regulated by the Wnt/β-catenin and transforming growth factor-β (TGF-β)/Smad pathways. **b** Relative luciferase activities of the TOP/FOP reporter or TGF-β reporter activity in the indicated cells. **c** Western blotting analysis of the expression of nuclear β-catenin, p-Smad2 (Ser465/467), and total Smad2 in the indicated cells. p84 and α-Tubulin were used as loading controls. **d**, **e** Predicted miR-455-3p targets (**d**) and RIP analysis of the association between miR-455-3p and the 3’UTR of the indicated targets (**e**). *GAPDH* served as a negative control. **f** Western blotting analysis of the expression of DKK3, GSK3β, Smurf2, and PPM1A in the indicated cells. α-Tubulin served as a loading control. Each bar represents the mean ± SD of three independent experiments. *P < 0.05

Consistent with the GSEA findings that miR-455-3p levels were significantly correlated with gene signatures regulated by the Wnt/β-catenin and TGF-β/Smad pathways (Fig. 5a and Additional file 5: Figure S4A), overexpression of miR-455-3p significantly enhanced, but silencing of miR-455-3p reduced, luciferase reporter activities and levels of expression of nuclear β-catenin, phosphorylated (p)-Smad2, and downstream genes in their respective pathways (Fig. 5b and c and Additional file 5: Figure S4B), suggesting that miR-455-3p contributes to activation of the Wnt/β-catenin and TGF-β/Smad pathways in ESCC. Furthermore, we found that miR-455-3p levels in 207 ESCC specimens were positively correlated with the expression of nuclear β-catenin and p-Smad2 (Additional file 5: Figure S4C). Meanwhile, the proportion of CD90^+^/CD271^+^ cells and tumorsphere-forming capability of miR-455-3p-transduced cells decreased via inhibition of the Wnt/β-catenin and TGF-β/Smad pathways upon treatment with their respective inhibitors (Additional file 5: Figure S4D and E), demonstrating that these two pathways are required for the miR-455-3p-induced T-IC traits of ESCC.

### miRNA-455-3p targets a number of negative regulators

Analysis using publicly available algorithms (Target Scan, miRWalk, and miRanda) showed that a number of negative regulators, including DKK3, GSK3β, TCF7L1, IGFBP4, AMER1, CSNK1A1, TLE3, TLE4, TCF3, NKD2, SOX1, and SOST (Wnt/β-catenin pathway) and Smurf2, NEDD4L, FKBP1A, BAMBI, PAK2, SKI, and PPM1A (TGF-β/Smad pathway), were potential targets of miR-455-3p (Fig. 5d). RIP assays revealed a higher degree of association of miR-455-3p with DKK3, GSK3β, Smurf2, and PPM1A in both EC-CR and Eca109 cells (Fig. 5e). The inhibitory effects of miR-455-3p on the protein expression and 3’UTRs-activity of these transcripts were verified in ESCC cells and further confirmed in 10 freshly collected ESCC specimens. These results suggest that miR-455-3p activates the Wnt/β-catenin and TGF-β/Smad pathways by directly binding to the 3′ UTRs of a number of negative regulators.

### Aberrant miRNA-455-3p expression contributes to the progression of various cancers

**Fig. 6:**
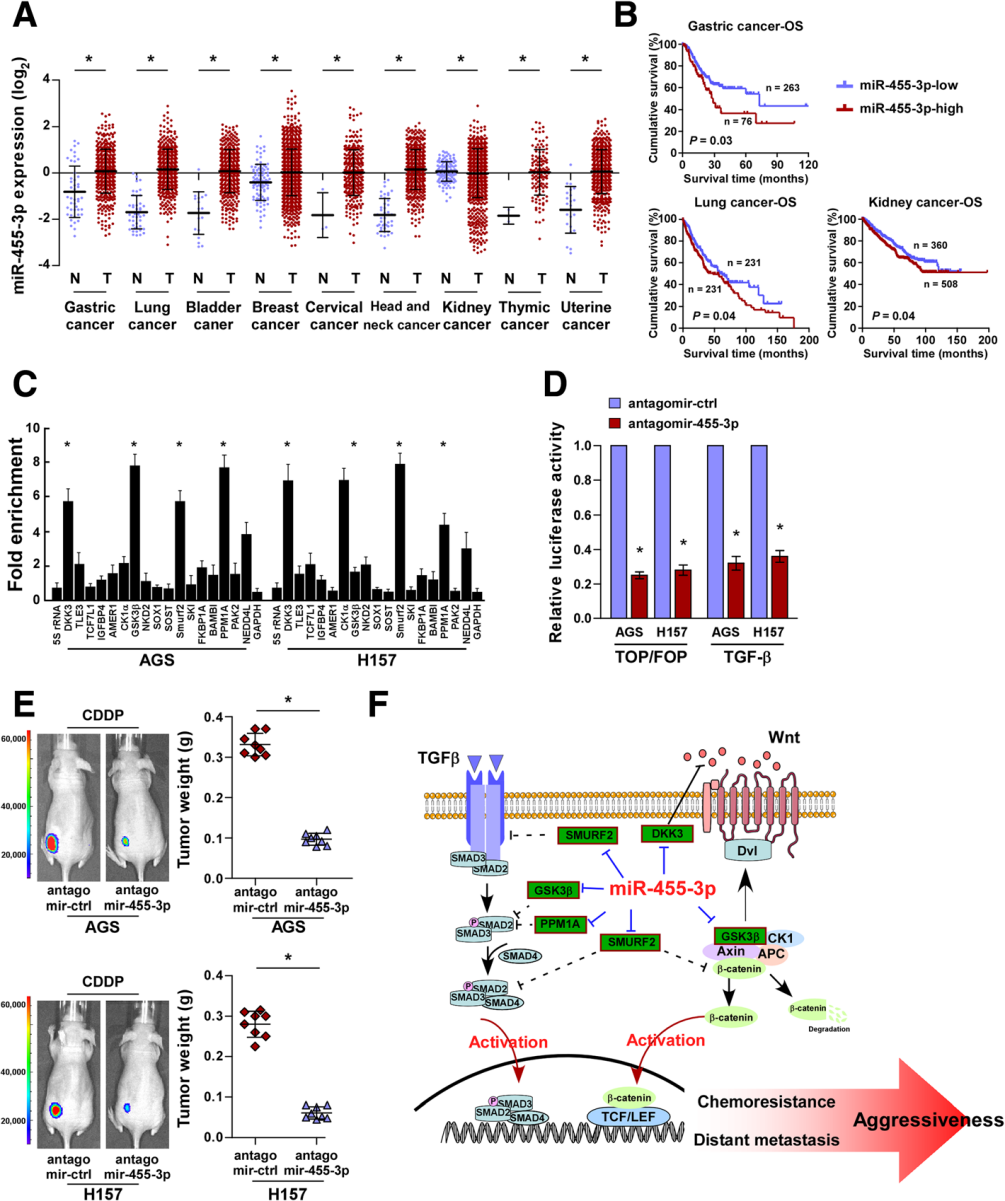
Aberrant miR-455-3p contributes to the progression of various cancers. **a** Analysis of TCGA datasets indicating that miR-455-3p is aberrantly upregulated in subsets of primary tumors, including gastric, lung, bladder, breast, cervical, head and neck, kidney, thyroid, and uterine cancers. **b** Kaplan–Meier analysis of overall survival for the indicated cancer patients with low or high miR-455-3p expression. **c** RIP analysis of the association between miR-455-3p and the 3’UTR of the indicated genes. *GAPDH* served as a negative control. **d** Relative luciferase activities of the TOP/FOP reporter or TGF-β reporter activity in the indicated cells. **e** Representative images of CDDP-treated tumor-bearing mice (*left panel*) and weight of xenografts (*right panel*) intratumorally injected with antagomir control or antagomirR-455-3p. **f** Hypothetical model illustrating that aberrant expression of miR-455-3p activates multiple cancer stem cell-associated signaling pathways, leading to cancer progression, chemotherapy failure, and poor clinical outcomes. Each bar represents the mean ± SD of three independent experiments. *P < 0.05

Analysis of TCGA datasets indicated that miR-455-3p was also markedly upregulated in other human cancers, including gastric, lung, bladder, breast, cervical, kidney, and uterine cancers (Fig. 6a), suggesting that miR-455-3p may also function as an oncomiR in other human cancers. Importantly, higher miR-455-3p expression was associated with shorter overall survival and significantly correlated with gene signatures regulated by the Wnt/β-catenin and TGF-β/Smad pathways in gastric, kidney, and lung cancers (Fig. 6b and Additional file 6: Figure S5). Furthermore, RIP assays indicated that miR-455-3p was associated with different negative regulators of the Wnt/β-catenin and TGF-β/Smad pathways in gastric and kidney cancer cells (Fig. 6c). Moreover, silencing miR-455-3p in gastric and bladder cancer cells dramatically decreased the transcriptional activities of the Wnt/β-catenin and TGF-β/Smad pathways and CDDP resistance (Fig. 6d and e). Altogether, these results imply that aberrant miR-455-3p expression activates T-IC-associated signaling pathways, leading to cancer progression, chemotherapy failure, and poor clinical outcomes (Fig. 6f).

## Discussion

It is generally acknowledged that an association exists between T-ICs and poor prognosis, tumor recurrence, and chemoradiotherapy failure in multiple human cancers [1–4]. Hence, the development of effective techniques for the enrichment and isolation of T-ICs, which are rare within tumors, has been a focus of cancer research over the past two decades. However, characterization of the biologic properties and regulatory mechanisms associated with T-ICs have been hampered by the difficulty of isolating T-IC populations. In this study, by employing a chemoresistant human ESCC patient-derived xenograft model, we successfully enriched chemoresistant ESCC cells that exhibited greater capacities for tumor-initiation and self-renewal. Furthermore, we demonstrated that miR-455-3p plays essential roles in ESCC chemoresistance and tumorigenesis, and that treatment with a miR-455-3p antagomir chemosensitizes ESCC cells and reduces ESCC T-ICs subpopulations. Our results suggest that the chemoresistant ESCC cells examined in our study possess T-IC-like traits, and that miR-455-3p represents a potential therapeutic target to achieve better clinical outcomes in cancer patients.

We found that the proportions of both CD90^+^ and CD271^+^ cells, previously identified as ESCC T-ICs [31, 32], were increased among chemoresistant ESCC cells, suggesting the existence of multiple T-IC subpopulations within ESCC tumors. Previously, two highly tumorigenic T-IC populations, the CD34 levels of which differ, were identified in skin squamous cell carcinoma [22]. The same phenomenon was also observed in liver (CD24^+^ and CD133^+^) and colon (CD44^+^ and CD26^+^) cancers [10, 12, 13, 35]. It was proposed that differentiated non-T-ICs can enter the T-IC state in response to TGF-β treatment, and that terminally differentiated neurons can revert to a stem cell-like state and express typical T-IC markers [36, 37]. This implies that the T-IC hierarchy is flexible and that cells can reversibly interconvert between the T-IC and non-T-IC states [38, 39]. We found that miR-455-3p overexpression significantly increased, but miR-455-3p inhibition reduced, the subpopulations of CD90^+^ and CD271^+^ T-ICs, suggesting that miR-455-3p functions in the interconversion between ESCC cells and ESCC T-IC subpopulations.

Recent advances have indicated that T-IC-targeting therapeutics represent a potentially effective strategy to improve the prognosis of patients suffering from deadly malignancies. T-IC-dependent pathways, such as Wnt/β-catenin and TGF-β/Smad signaling [16–21], are emerging as attractive targets because their inactivation enables the elimination of T-ICs. However, blockade of a single signaling pathway required by a T-IC subpopulation may be insufficient to eradicate the entire T-IC population because heterogeneous populations of T-ICs exist within tumors and multiple signaling pathways collaborate in their maintenance. Therefore, the identification of targets that simultaneously regulate multiple T-IC-associated pathways may represent a more promising approach. We found that silencing of miR-455-3p simultaneously deactivated multiple T-IC-associated pathways, resulting in functional inhibition of ESCC chemoresistance and tumor recurrence, suggesting that miR-455-3p may be a suitable therapeutic target for the treatment of ESCC. Our findings provide an attractive therapeutic approach to achieve better clinical outcomes in cancer patients.

Consistent with our finding that miR-455-3p is upregulated in ESCC and multiple distinct cancer types, miR-455-3p is also overexpressed in glioma, oral squamous cell cancer, and triple-negative breast cancer, where it contributes to cancer chemoresistance, proliferation, and invasion/migration [40–42]. However, miR-455-3p is reportedly downregulated in prostate and colon cancer, and upregulation of miR-455-3p can inhibit the cancer proliferation [43, 44]. These studies imply that miR-455-3p can act as either an oncomiR or a tumor-suppressive miRNA depending on the tumor type. To explore the molecular mechanism underlying the function of miR-455-3p in ESCC chemoresistance, we examined miR-455-3p expression in ESCC and found that miR-455-3p levels are significantly correlated with the clinical features and overall/relapse-free survival of patients with ESCC, suggesting that miR-455-3p may be associated with chemotherapy failure in these patients. Furthermore, we demonstrated that aberrantly expressed miR-455-3p in ESCC cells simultaneously activates Wnt/β-catenin and TGF-β/Smad signaling through concurrent suppression of multiple negative regulators of these pathways. Therefore, our findings not only present a novel mechanism by which the Wnt/β-catenin and TGF-β/Smad signaling pathways are constitutively active in ESCC, but also highlight the significant contribution of these pathways to T-IC traits.

## Conclusions

In this study, we identified miR-455-3p as essential for ESCC chemoresistance both in vivo and in vitro. We found that miR-455-3p levels are significantly correlated with poorer disease-free survival and overall survival in patients with primary ESCC. Inhibition of miR-455-3p chemosensitizes ESCC cells and reduces the subpopulations of CD90^+^ and CD271^+^ T-ICs via the suppression of multiple T-IC-associated pathways, including the Wnt/β-catenin and TGF-β pathways. Importantly, miR-455-3p is aberrantly upregulated in numerous cancers and significantly associated with the decreased overall survival of cancer patients. Our findings provide an attractive therapeutic approach for targeting T-ICs to achieve better clinical outcomes in cancer patients.

## Additional files

Additional file 1: Supplemental information. (DOCX 42 kb)

Additional file 2: Figure S1. miR-455-3p enhances ESCC chemoresistance and promotes ESCC tumorgencity. (**A**) GSEA of TCGA datasets indicating that miR-455-3p expression was significantly correlated with chemoresistance gene signatures. (**B**) The apoptotic ratio of the indicated cells treated with CDDP (20 μM) or DOC (1.5 nM) for 24 h. (**C**) Images (left) and weight (upper right) of xenografts and apoptotic ratio (lower right) of the indicated tumors. (**D**) GSEA analysis indicating miR-455-3p expression was significantly associated with stem cell-like traits. (**E**) Representative images (left) and quantification (right) of tumorspheres formed by the indicated cells. (**F**) Flow cytometry analysis of the percentages of the CD90^+^/CD271^+^ subpopulations (left) and SP cells (right) of the indicated cells. Each bar represents the mean ± SD of three independent experiments. * *P* < 0.05. (TIFF 1054 kb)

Additional file 3: Figure S2. Silencing miR-455-3p chemosensitizes ESCC cells and reduces stem cell-like traits of ESCC. (**A**) Quantification of TUNEL-stained cells (apoptotic ratio) in the indicated tumors. (**B**) Representative tumor growth curves of xenografts derived from EC-CR cells co-treated with CDDP (5 mg/kg) and antagomir-control or with CDDP (5 mg/kg) and antagomir-455-3p on the indicated days (left) and apoptotic ratio (right) of the indicated tumors. (**C**) Representative images (left) and quantification (right) of tumorspheres formed by the indicated cells. (**D**, **E**) Flow cytometry analysis of the percentages of CD90^+^/CD271^+^ subpopulations (D) and SP cells (E) of the indicated cells. (**F**) Real-time PCR analysis of the mRNA expression of the indicated transcripts in miR-455-3p-silenced EC-CR and Eca109 cells. Each bar represents the mean ± SD of three independent experiments. * *P* < 0.05. (TIFF 452 kb)

Additional file 4: Figure S3. miR-455-3p overexpression correlates with poor prognosis in ESCC patient. (**A**) Analysis of TCGA datasets indicating that miR-455-3p was significantly upregulated in 13 pairs of ESCC samples (T) compared with adjacent normal tissues (ANT; *P =* 0.001). (**B**) Kaplan–Meier analysis of overall and disease-free survival curves for patients with ESCC exhibiting low or high miR-455-3p expression in TCGA datasets. (**C**) Real-time PCR analyses of miR-433-5p expression in PDEC1 and PDEC2 cells. Transcript levels were normalized to *U6* expression. (**D**) Flow cytometry analysis of the percentage of SP cells among the indicated cells. Each bar represents the mean ± SD of three independent experiments. *P < 0.05. (TIFF 293 kb)

Additional file 5: Figure S4. miR-455-3p overexpression activates T-IC-associated signaling pathways. (**A**) GSEA analysis of TCGA datasets indicating that miR-455-3p expression was significantly correlated with the gene signatures regulated by the Wnt/β-catenin and TGF-β/Smad pathways. (**B**) Heat map showing real-time PCR results of the downstream target genes of either Wnt/β-catenin or TGF-β signaling in the indicated cells, as compared with corresponding control cells. Pseudo- color scale values were Log2 transformed. (**C**) miR-455-3p levels were positively correlated with the expression of nuclear β-catenin and p-Smad2 (Ser465/467) in 207 primary human ESCC specimens. Left: Two representative cases are shown. Scale bar: 50 μm. Right: The percentages of specimens showing low or high miR-455-3p expression relative to levels of nuclear β-catenin and p-Smad2 (Ser465/467). (**D**, **E**) Quantification of CD90^+^/CD271^+^ subpopulations (**D**) and number of tumorspheres (**E**) in the indicated cells treated with a β-catenin inhibitor or TGF-β inhibitor. (**F**) Luciferase assay of the indicated cells transfected with the pGL3-DKK3 (−GSK3β, −Smurf2, −PPM1A) reporter with miR-455-3p mimic, miR-455-3p antagomir or miR-455-3p-mut mimic. (**G**) Correlation analysis of miR-455-3p with nuclear β-catenin, p-Smad2 (Ser465/467), DKK3, GSK3β, Smurf2, and PPM1A in 10 freshly collected human ESCC samples. Each bar represents the mean ± SD of three independent experiments. *P < 0.05. (TIFF 1465 kb)

Additional file 6: Figure S5. GSEA analysis of TCGA datasets indicating that miR-455-3p levels are correlated with the gene signatures of the Wnt/β-catenin and TGF-β/Smad pathways in gastric and lung cancers. (TIFF 258 kb)

## Abbreviations

AML: Acute myeloid leukemia
CSCs: Cancer stem cells
DKK3: Dickkopf WNT signaling pathway inhibitor 3
DOC: Docetaxel
EC-CR: CDDP-resistant ESCC
EC-UT: CDDP-untreated ESCC
ESCC: Esophageal squamous cell carcinoma
GICs: Glioma-initiating cells
GSEA: Gene set enrichment analysis
GSK3β: Glycogen synthase kinase 3β
HCC: Hepatocellular carcinoma
miR-455-3p: microRNA-455-3p
miRNAs: microRNAs
NOG: NOD/Shi-scid/IL-2Rγnull
PDEC: Patient-derived esophageal squamous cell carcinoma
PDX: Patient-derived xenograft
PPM1A: Protein phosphatase, Mg2+/Mn2+ dependent 1A
RIP: RNA immunoprecipitation
SCCs: Squamous cell carcinomas
Smurf2: SMAD specific E3 ubiquitin protein ligase 2
SP: Side-population
STR: Short tandem repeat
TCGA: The Cancer Genome Atlas
TGF-β: Transforming growth factor-β
T-ICs: Tumor-initiating cells
