# Correction to: Antagonizing miR-455-3p inhibits chemoresistance and aggressiveness in esophageal squamous cell carcinoma

**DOI:** 10.1186/s12943-021-01425-4

**Published:** 2021-12-01

**Authors:** Aibin Liu, Jinrong Zhu, Geyan Wu, Lixue Cao, Zhanyao Tan, Shuxia Zhang, Lili Jiang, Jueheng Wu, Mengfeng Li, Libing Song, Jun Li

**Affiliations:** 1 Program of Cancer Research, Affiliated Guangzhou Women and Children’s Hospital, Department of Biochemistry, Zhongshan School of Medicine, Sun Yat-sen University, 74 Zhongshan Road II, Guangzhou, 510080 Guangdong China; 2 State Key Laboratory of Oncology in Southern China, Department of Experimental Research, Cancer Center, Sun Yat-sen University, Guangzhou, 510060 China; 3 Key Laboratory of Protein Modification and Degradation, School of Basic Medical Sciences, Affiliated Cancer Hospital & Institute of Guangzhou Medical University, Guangzhou, China; 4 Department of Microbiology, Zhongshan School of Medicine, Sun Yat-sen University, Guangzhou, China

**Correction to: Mol Cancer 16, 106 (2017)**

**https://doi.org/10.1186/s12943-017-0669-9**

Following the publication of the original paper [1], the authors found an error in Supplemental Figure 1E. The correct sphere image has been used in the updated figure presented here.

This correction does not affect the description, interpretation, or conclusions of the manuscript. The original article has been updated.

## Supplementary Information

**Additional file 1: Supplementary Figure S1**.
