# Supplementary material for: Antagonizing miR-455-3p inhibits chemoresistance and aggressiveness in esophageal squamous cell carcinoma
Source: Mol Cancer. 2017 Jun 21;16:106. doi: 10.1186/s12943-017-0669-9 (PMC5479030; doi:10.1186/s12943-017-0669-9)
Supplement: Supplementary file 1 — Supplemental information. (DOCX 42 kb) [file 12943_2017_669_MOESM1_ESM.docx]

**Supplemental Information**

**Supplementary Material and methods**

**RNA Extraction and Real-Time Quantitative PCR.** Total mRNA and miRNA from cultured cells and fresh surgical ESCC tissues was extracted using the mirVana miRNA Isolation Kit (Ambion, Austin, TX, USA) according to the manufacturer’s manual. The expression level of miR-455-3p was quantified using miRNA-specific TaqMan MiRNA Assay Kit (Applied Biosystems). Real-time PCR was performed using the Applied Biosystems 7500 Sequence Detection system. The expression of miRNA was defined based on the threshold cycle (Ct), and relative expression levels were calculated as 2^-[(Ct of^ *^miR-455-3p^*^) – (Ct of^ *^U6^*^)]^ after normalization with reference to the quantification of *U6* small nuclear RNA expression. Amplification and quantification of cDNA was performed using an ABI Prism 7500 Sequence Detection System (Applied Biosystems) and SYBR Green I dye (Molecular Probes, Eugene, Oregon, USA). Relative gene expression was evaluated by the threshold cycle (Ct) method, normalized to the housekeeping gene *GAPDH*, according to the formula 2^-[(Ct of^ *^gene^*^) – (Ct of^ *^GAPDH^*^)]^.

**Primers and Oligonucleotides**.

Cloning primers:

| miR-455-forward | GCCGGATCCAGTCGTGTTGCTTTATCTACAGAGC |
| --- | --- |
| miR-455-reverse | GCCGAATTCTCAGAGCGGTCAAGCAGTGA |
| DKK3-3’UTR-luc-forward | GCCGGATCCAGGTTGTTTCTATTTTACTTTTTCTCC |
| DKK3-3’UTR-luc-reverse | GCCCTCGAGCAGGCTGTGGGTAGATGTGC |
| GSK3β-3’UTR-luc-forward | GCCGGATCCACTGATTGTGCCTTAAAGATTCATA |
| GSK3β-3’UTR-luc-reverse | GCCCTCGAGGAAAAACCACCAGTTACTTGAGTG |
| SMURF2-3’UTR-luc-forward | GCCGGATCCAACTGGTTTTTTCTTGTCTTCCCT |
| SMURF2-3’UTR-luc-reverse | GCCCTCGAGACTTCGGCATGGGCACATAC |
| PPM1A-3’UTR-luc-forward | GCCGGATCCATCTGTGGTAGAGTGCGAAGTATGA |
| PPM1A-3’UTR-luc-reverse | GCCCTCGAGCTTAAACTCAAACCCCACCTACATT |

Real-time PCR primers:

| SOX2-forward | GCTTAGCCTCGTCGATGAAC |
| --- | --- |
| SOX2-reverse | AACCCCAAGATGCACAACTC |
| OCT4-forward | GGTTCTCGATACTGGTTCGC |
| OCT4-reverse | GTGGAGGAAGCTGACAACAA |
| ALDH1-forward | CCACTCACTGAATCATGCCA |
| ALDH1-reverse | GCACGCCAGACTTACCTGTC |
| BMI1-forward | TCGTTGTTCGATGCATTTCT |
| BMI1-reverse | CTTTCATTGTCTTTTCCGCC |
| ABCG2-forward | TGGTGTTTCCTTGTGACACTG |
| ABCG2-reverse | TGAGCCTTTGGTTAAGACCG |
| CD90-forward | GGGAGACCTGCAAGACTGTT |
| CD90-reverse | CGGAAGACCCCAGTCCA |
| CD271-forward | CAGGCTTTGCAGCACTCAC |
| CD271-reverse | CTGCTGCTGTTGCTGCTTCT |
| GAPDH-forward | AATGAAGGGGTCATTGATGG |
| GAPDH-reverse | AAGGTGAAGGTCGGAGTCAA |

Real-time PCR primers for RIP:

| DKK3-RIP-forward | CGACTGCGAACACTGAACTC |
| --- | --- |
| DKK3-RIP-reverse | AAGAACTCTGGATGAATACATGGTG |
| TLE3-RIP-forward | GGTCTCTCTTTGCCGATTGT |
| TLE3-RIP-reverse | GGCCTCCTCTCCCACTGAT |
| TCF7L1-RIP-forward | TGATTCAGAAGAAAAAGAAAAAGGA |
| TCF7L1-RIP-reverse | TGCTGTTGTTACCAGCCACT |
| IGFBP4-RIP-forward | AGGGAGGTGGGGTACATTTC |
| IGFBP4-RIP-reverse | CCTAGCACGTGGGTCTTCTC |
| AMER1-RIP-forward | GTCCCCTTTCCCCAAGAAG |
| AMER1-RIP-reverse | CCTATGCAAGGGTTTCCTCA |
| CK1a-RIP-forward | GCCAAGAACCAAGTGACGTT |
| CK1a-RIP-reverse | CCACCTCCTTGATCTAACACTG |
| GSK3b-RIP-forward | ACTACTTGAATGCCTCTGTGACTG |
| GSK3b-RIP-reverse | AAAAACATTGAGCATACTTTTCACA |
| NKD2-RIP-forward | CAGGCTGTGGACTGCTACTG |
| NKD2-RIP-reverse | TCAGGCACTCATGCTCACTC |
| SOX1-RIP-forward | AAAAACACTTGAAGCCCAGATG |
| SOX1-RIP-reverse | AAGGAAGGGTGTTGAGAAAGC |
| SOST-RIP-forward | AAGTTTTAAACAGAAGCACATGACA |
| SOST-RIP-reverse | CAAGTCCCACGTGGAAGAAT |
| SMURF2-RIP-forward | AAATCTGGGCTTTGAATTTCC |
| SMURF2-RIP-reverse | CCCAAACTAAGGATGGTTGG |
| SKI-RIP-forward | GCTCCCTTCATTTTGGGACT |
| SKI-RIP-reverse | CGTTTAAATGAACGAACAAAAA |
| FKBP1A-RIP-forward | GCTGCAAAGCCATAGCAGAT |
| FKBP1A-RIP-reverse | GGGCTTTTAATTTAACCCAAAGA |
| BAMBI-RIP-forward | GAAATCAGTGAACCCCTTCCT |
| BAMBI-RIP-reverse | ATCAAGTGGAGAGGCTGCAT |
| NEDD4L-RIP-forward | TTCAGGCATTGGGGTACATC |
| NEDD4L-RIP-reverse | CAAAGTGGAGTTTATAAGGATTTGTTT |
| PAK2-RIP-forward | CCCCTTCTTTAGGGTCCAGA |
| PAK2-RIP-reverse | CACATGATAAATGGCCCTGA |
| PPM1A-RIP-forward | AAAGTTAAAGAGTAATTCAGAAGAAAA |
| PPM1A-RIP-reverse | TCAAGGATACTGAATGGGCTTA |
| GAPDH-RIP-forward | CAGCAAGAGCACAAGAGGAA |
| GAPDH-RIP-reverse | CTGTGAGGAGGGGAGATTCA |

**Cell Treatments.** Cisplatin (20 μM; Selleck Chemicals, Houston, TX) or Docetaxel (1.5 nM; Selleck Chemicals, Houston, TX) were dissolved in dimethyl sulfoxide and incubated indicated cells for 24 hours. β-catenin/TCF Inhibitor FH535 (20 μM; Selleck Chemicals, Houston, TX), TGF-β inhibitor LY2109761 (10 μM; Selleck Chemicals, Houston, TX) were dissolved in dimethyl sulfoxide and incubated indicated cells for 48 hours.

**Western blotting analysis (WB).** WB was performed using anti-Smad2, anti-p-Smad2 (Ser465/467), anti-β-catenin, anti-Smurf2, and anti-PPM1A, antibodies (Cell Signaling, Danvers, MA); anti-DKK3 and anti-GSK3β antibodies (Sigma-Aldrich, Germany). To control sample loading, the blotting membranes were stripped and re-probed with anti-α-tubulin or anti-p84 antibodies (Sigma, Saint Louis, MO).

**Immunohistochemistry.** Immunohistochemistry (IHC) analysis was performed on the 207 paraffin-embedded ESCC tissue sections. The degree of immunostaining of formalin-fixed, paraffin-embedded sections were reviewed and scored separately by two independent pathologists. The scores were determined by combining the proportion of positively-stained tumor or normal esophageal epithelial cells and the intensity of staining. Cell proportions were scored as follows: 0, no positive cells; 1, <10% positive cells; 2, 10%-35% positive cells; 3, 35%-75% positive cells; 4, >75% positive cells. Staining intensity was graded according to the following standard: 1, no staining; 2, weak staining (light yellow); 3, moderate staining (yellow brown); 4, strong staining (brown). The staining index (SI) was calculated as the product of the staining intensity score and the proportion of positive cells. Using this method of assessment, we evaluated protein expression in benign esophageal epithelia and malignant lesions by determining the SI, with possible scores of 0, 2, 3, 4, 6, 8, 9, 12, and 16. Samples with a SI ≥ 8 were determined as high expression and samples with a SI < 8 were determined as low expression. Cutoff values were determined on the basis of a measure of heterogeneity using the log-rank test with respect to overall survival.

**Tumoresphere formation assay.** Cells (2 × 10^3^) were seeded in 6-well ultra-low cluster plates and 10 or 20 cells were seeded in 24-well ultra-low cluster plates (Corning, NY) for 10 days. Spheres were cultured in DMEM/F12 serum-free medium (Invitrogen, Grand Island, NY) supplemented with 2% B27 (Invitrogen, Grand Island, NY), 20 ng/ml of EGF, and 20 ng/ml of bFGF (PeproTech, Offenbach, Germany), 0.4% bovine serum albumin (BSA) (Sigma, St. Louis, MO, USA), and 5 μg/ml insulin.

**Flow cytometry analysis.** Cells were dissociated with trypsin and re-suspended at 1 × 10^6^ cells/ml in DMEM containing 2% fetal bovine serum (FBS) and then pre-incubated at 37°C for 30 min with or without 100 μM verapamil (Sigma-Aldrich, Germany) to inhibit ABC transporters. The cells were subsequently incubated for 90 min at 37°C with 5 μg/ml Hoechst 33342 (Sigma-Aldrich, Germany). Finally the cells were incubated on ice for 10 min and washed with ice-cold PBS before flow cytometry analysis. The data were analyzed by Summit5.2 (Beckman Coulter, Indianapolis, IN).

**Luciferase assay.** Cells (1 × 10^4^) were seeded in triplicate in 48-well plates and allowed to settle for 24 h. One hundred nanograms of luciferase reporter plasmids or the control plasmid, plus 1 ng of pRL-TK renilla plasmid (Promega), were transfected into cells using the Lipofectamine 3000 reagent (Invitrogen) according to the manufacturer’s instruction. Luciferase and renilla signals were measured using the Dual Luciferase Reporter Assay Kit (Promega) according to a protocol provided by the manufacturer.

**Figure S1. miR-455-3p enhances ESCC chemoresistance and promotes ESCC tumorgencity.** (**A**) GSEA of TCGA datasets indicating that miR-455-3p expression was significantly correlated with chemoresistance gene signatures. (**B**) The apoptotic ratio of the indicated cells treated with CDDP (20 μM) or DOC (1.5 nM) for 24 h. (**C**) Images (left) and weight (upper right) of xenografts and apoptotic ratio (lower right) of the indicated tumors. (**D**) GSEA analysis indicating miR-455-3p expression was significantly associated with stem cell-like traits. (**E**) Representative images (left) and quantification (right) of tumorspheres formed by the indicated cells. (**F**) Flow cytometry analysis of the percentages of the CD90^+^/CD271^+^ subpopulations (left) and SP cells (right) of the indicated cells. Each bar represents the mean ± SD of three independent experiments. * *P* <0.05.

**Figure S2. Silencing miR-455-3p chemosensitizes ESCC cells and reduces stem cell-like traits of ESCC.** (**A**) Quantification of TUNEL-stained cells (apoptotic ratio) in the indicated tumors. (**B**) Representative tumor growth curves of xenografts derived from EC-CR cells co-treated with CDDP (5 mg/kg) and antagomir-control or with CDDP (5 mg/kg) and antagomir-455-3p on the indicated days (left) and apoptotic ratio (right) of the indicated tumors. (**C**) Representative images (left) and quantification (right) of tumorspheres formed by the indicated cells. (**D**, **E**) Flow cytometry analysis of the percentages of CD90^+^/CD271^+^ subpopulations (D) and SP cells (E) of the indicated cells. (**F**) Real-time PCR analysis of the mRNA expression of the indicated transcripts in miR-455-3p-silenced EC-CR and Eca109 cells. Each bar represents the mean ± SD of three independent experiments. * *P* <0.05.

**Figure S3．miR-455-3p overexpression correlates with poor prognosis in ESCC patient.** (**A**) Analysis of TCGA datasets indicating that miR-455-3p was significantly upregulated in 13 pairs of ESCC samples (T) compared with adjacent normal tissues (ANT; *P =* 0.001). (**B**) Kaplan–Meier analysis of overall and disease-free survival curves for patients with ESCC exhibiting low or high miR-455-3p expression in TCGA datasets. (**C**) Real-time PCR analyses of miR-433-5p expression in PDEC1 and PDEC2 cells. Transcript levels were normalized to *U6* expression. (**D**) Flow cytometry analysis of the percentage of SP cells among the indicated cells. Each bar represents the mean ± SD of three independent experiments. **P <*0.05.

**Figure S4. miR-455-3p overexpression activates T-IC-associated signaling pathways.** (**A**) GSEA analysis of TCGA datasets indicating that miR-455-3p expression was significantly correlated with the gene signatures regulated by the Wnt/β-catenin and TGF-β/Smad pathways. (**B**) Heat map showing real-time PCR results of the downstream target genes of either Wnt/β-catenin or TGF-β signaling in the indicated cells, as compared with corresponding control cells. Pseudo- color scale values were Log2 transformed. (**C**) miR-455-3p levels were positively correlated with the expression of nuclear β-catenin and p-Smad2 (Ser465/467) in 207 primary human ESCC specimens. Left: Two representative cases are shown. Scale bar: 50 μm. Right: The percentages of specimens showing low or high miR-455-3p expression relative to levels of nuclear β-catenin and p-Smad2 (Ser465/467). (**D**, **E**) Quantification of CD90^+^/CD271^+^ subpopulations (**D**) and number of tumorspheres (**E**) in the indicated cells treated with a β-catenin inhibitor or TGF-β inhibitor. (**F**) Luciferase assay of the indicated cells transfected with the pGL3-DKK3 (-GSK3β, -Smurf2, -PPM1A) reporter with miR-455-3p mimic, miR-455-3p antagomir or miR-455-3p-mut mimic. (**G**) Correlation analysis of miR-455-3p with nuclear β-catenin, p-Smad2 (Ser465/467), DKK3, GSK3β, Smurf2, and PPM1A in 10 freshly collected human ESCC samples. Each bar represents the mean ± SD of three independent experiments. **P <*0.05.

**Figure S5. GSEA analysis of TCGA datasets indicating that miR-455-3p levels are correlated with the gene signatures of the Wnt/β-catenin and TGF-β/Smad pathways in gastric and lung cancers.**

**Supplementary Tables**

**Table S1**. Clinicopathological characteristics of studied patients and expression of miR-455-3p in ESCC

| **Factor** | **No.** | **(%)** |
| --- | --- | --- |
| **Gender** |  |  |
| Male | 161 | 77.8 |
| Female | 46 | 22.2 |
| **Age (years)** |  |  |
| ≤57 | 104 | 50.2 |
| >57 | 103 | 49.8 |
| **Clinical stage** |  |  |
| I | 30 | 14.5 |
| II | 88 | 42.5 |
| III | 67 | 32.4 |
| IV | 22 | 10.6 |
| **T classification** |  |  |
| T_1_ | 33 | 15.9 |
| T_2_ | 46 | 22.2 |
| T_3_ | 119 | 57.5 |
| T_4_ | 9 | 4.4 |
| **N classification** |  |  |
| N_0_ | 99 | 47.8 |
| N_1_ | 104 | 50.2 |
| N_2_ | 4 | 2.0 |
| **M classification** |  |  |
| No | 183 | 88.4 |
| Yes | 24 | 11.6 |
| **Histological differentiation** |  |  |
| Well | 63 | 30.4 |
| Moderate | 83 | 40.1 |
| Poor | 61 | 29.5 |
| **Vital status** |  |  |
| Alive | 85 | 41.1 |
| Dead | 122 | 58.9 |
| **Expression of miR-455-3p** |  |  |
| Low expression | 103 | 49.8 |
| High expression | 104 | 50.2 |

**Table S2**. Correlation between the clinicopathological features and expression of miR-455-3p

| **Patient characteristics** | | | **miR-455-3p expression** | | ***P*-value** |
| --- | --- | --- | --- | --- | --- |
|  |  |  | **Low** | **High** |  |
| **Gender** | | Male | 75 | 86 | 0.097 |
|  |  | Female | 28 | 18 |  |
| **Age (years)** | | ≤57 | 59 | 45 | 0.052 |
|  |  | >57 | 44 | 59 |  |
| **Clinical stage** | I | 25  132  62 | 5  44 | < 0.001 |  |
|  | II | 61 | 27 |  |  |
|  | III | 13 | 54 |  |  |
|  | IV | 4 | 18 |  |  |
| **T classification** | T_1_ | 25  64  106 | 8  18 | < 0.001 |  |
|  | T_2_ | 31 | 15 |  |  |
|  | T_3_ | 44 | 75 |  |  |
|  | T_4_ | 3 | 6 |  |  |
| **N classification** | N_0_ | 73  204 | 26  0 | < 0.001 |  |
|  | N_1_ | 29 | 75 |  |  |
|  | N_2_ | 1 | 3 |  |  |
| **M classification** | No | 97 | 86 | 0.01 |  |
|  | Yes | 6 | 18 |  |  |
| **Histological**  **differentiation** | Well | 38  174 | 25 | 0.023 |  |
|  | Moderate | 43 | 40 |  |  |
|  | Poor | 22 | 39 |  |  |
| **Vital status** | Alive | 59 | 26 | < 0.001 |  |
|  | Dead | 44 | 78 |  |  |

**Table S3**. Univariate and multivariate analysis of different prognostic parameters in patients with ESCC by Cox-regression analysis

|  | **Univariate analysis** | **Multivariate analysis** | | |
| --- | --- | --- | --- | --- |
|  | ***P*** | ***P*** | **Hazard ratio** | **95% CI** |
| **T classification** | < 0.001 | 0.023 | 1.503 | 1.059-2.134 |
| T_1_ |  |  |  |  |
| T_2_ |  |  |  |  |
| T_3_ |  |  |  |  |
| T_4_ |  |  |  |  |
| **N classification** | < 0.001 | 0.019 | 1.835 | 1.103-3.052 |
| N_0_ |  |  |  |  |
| N_1_ |  |  |  |  |
| **M classification** | < 0.001 | 0.036 | 1.767 | 1.038-3.008 |
| M_0_ |  |  |  |  |
| M_1_ |  |  |  |  |
| **miR-455-3p expression** | < 0.001 | < 0.001 | 1.674 | 1.480-1.893 |
| Low expression |  |  |  |  |
| High expression |  |  |  |  |
