# Supplementary figures and images for: Antagonizing miR-455-3p inhibits chemoresistance and aggressiveness in esophageal squamous cell carcinoma
Source: Mol Cancer. 2017 Jun 21;16:106. doi: 10.1186/s12943-017-0669-9 (PMC5479030; doi:10.1186/s12943-017-0669-9)

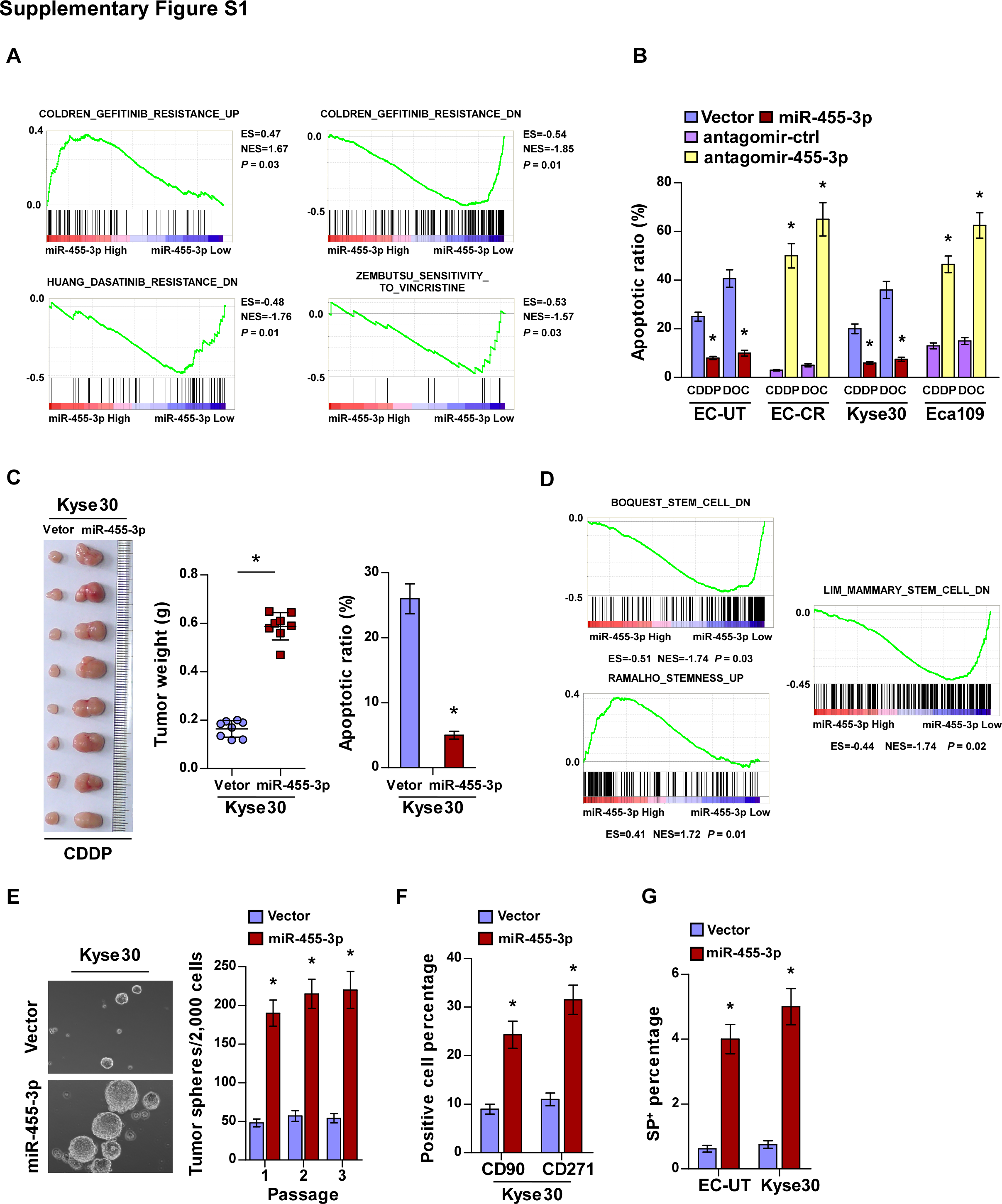

Supplement: Supplementary file 2 — miR-455-3p enhances ESCC chemoresistance and promotes ESCC tumorgencity. (A) GSEA of TCGA datasets indicating that miR-455-3p expression was significantly correlated with chemoresistance gene signatures. (B) The apoptotic ratio of the indicated cells treated with CDDP (20 μM) or DOC (1.5 nM) for 24 h. (C) Images (left) and weight (upper right) of xenografts and apoptotic ratio (lower right) of the indicated tumors. (D) GSEA analysis indicating miR-455-3p expression was significantly associated with stem cell-like traits. (E) Representative images (left) and quantification (right) of tumorspheres formed by the indicated cells. (F) Flow cytometry analysis of the percentages of the CD90+/CD271+ subpopulations (left) and SP cells (right) of the indicated cells. Each bar represents the mean ± SD of three independent experiments. * P < 0.05. (TIFF 1054 kb) [file 12943_2017_669_MOESM2_ESM.tif]

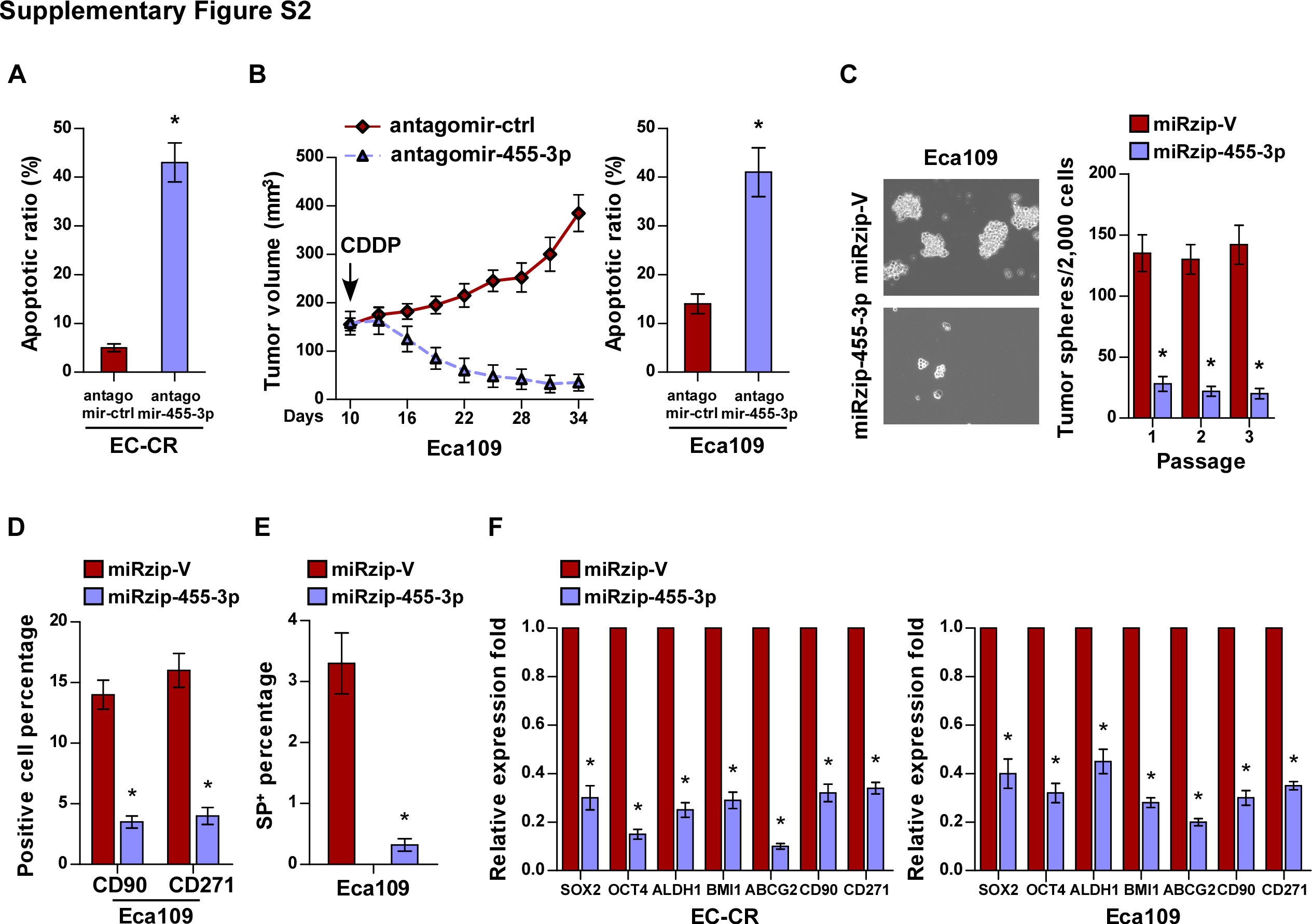

Supplement: Supplementary file 3 — Silencing miR-455-3p chemosensitizes ESCC cells and reduces stem cell-like traits of ESCC. (A) Quantification of TUNEL-stained cells (apoptotic ratio) in the indicated tumors. (B) Representative tumor growth curves of xenografts derived from EC-CR cells co-treated with CDDP (5 mg/kg) and antagomir-control or with CDDP (5 mg/kg) and antagomir-455-3p on the indicated days (left) and apoptotic ratio (right) of the indicated tumors. (C) Representative images (left) and quantification (right) of tumorspheres formed by the indicated cells. (D, E) Flow cytometry analysis of the percentages of CD90+/CD271+ subpopulations (D) and SP cells (E) of the indicated cells. (F) Real-time PCR analysis of the mRNA expression of the indicated transcripts in miR-455-3p-silenced EC-CR and Eca109 cells. Each bar represents the mean ± SD of three independent experiments. * P < 0.05. (TIFF 452 kb) [file 12943_2017_669_MOESM3_ESM.tif]

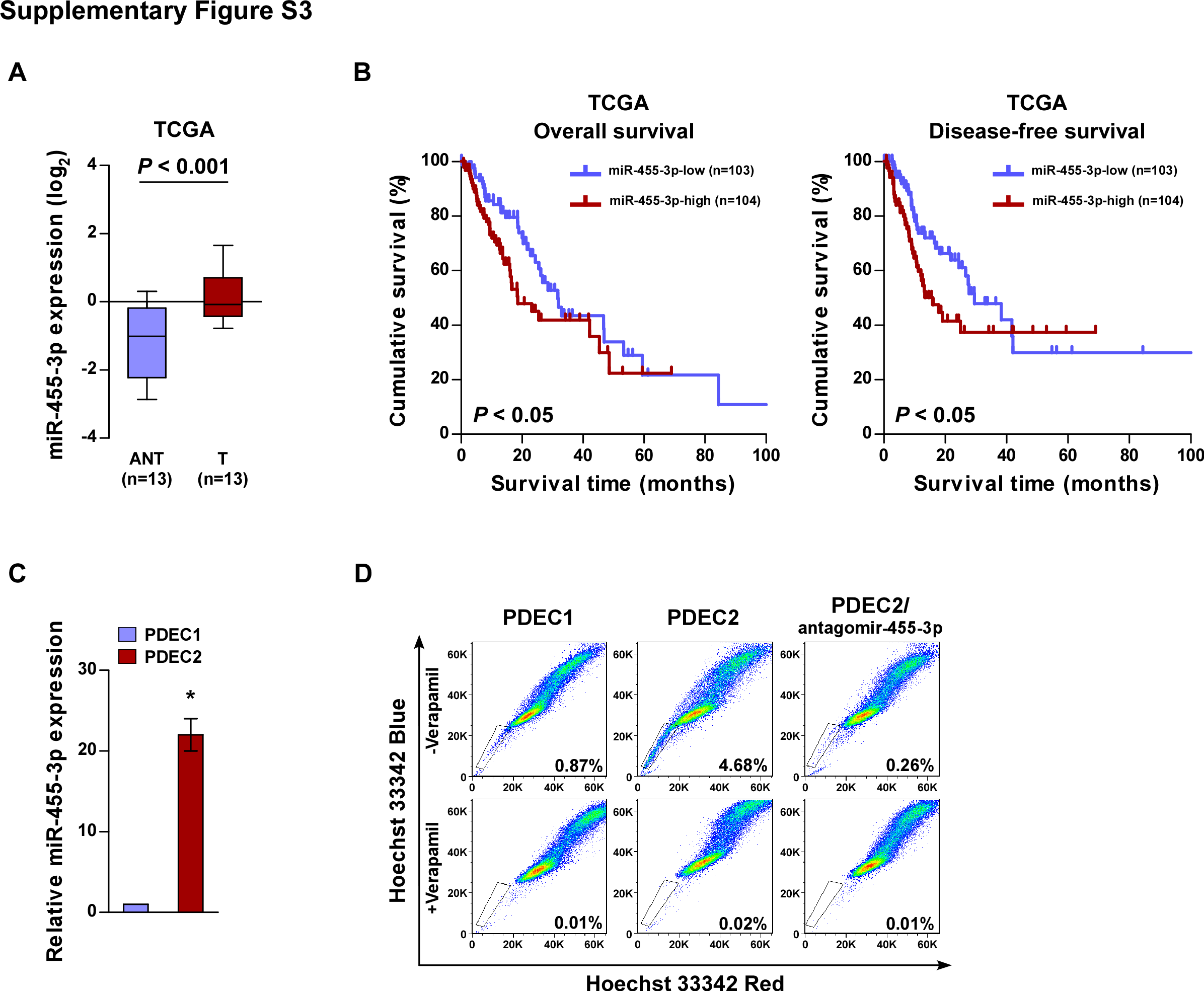

Supplement: Supplementary file 4 — miR-455-3p overexpression correlates with poor prognosis in ESCC patient. (A) Analysis of TCGA datasets indicating that miR-455-3p was significantly upregulated in 13 pairs of ESCC samples (T) compared with adjacent normal tissues (ANT; P = 0.001). (B) Kaplan–Meier analysis of overall and disease-free survival curves for patients with ESCC exhibiting low or high miR-455-3p expression in TCGA datasets. (C) Real-time PCR analyses of miR-433-5p expression in PDEC1 and PDEC2 cells. Transcript levels were normalized to U6 expression. (D) Flow cytometry analysis of the percentage of SP cells among the indicated cells. Each bar represents the mean ± SD of three independent experiments. *P < 0.05. (TIFF 293 kb) [file 12943_2017_669_MOESM4_ESM.tif]

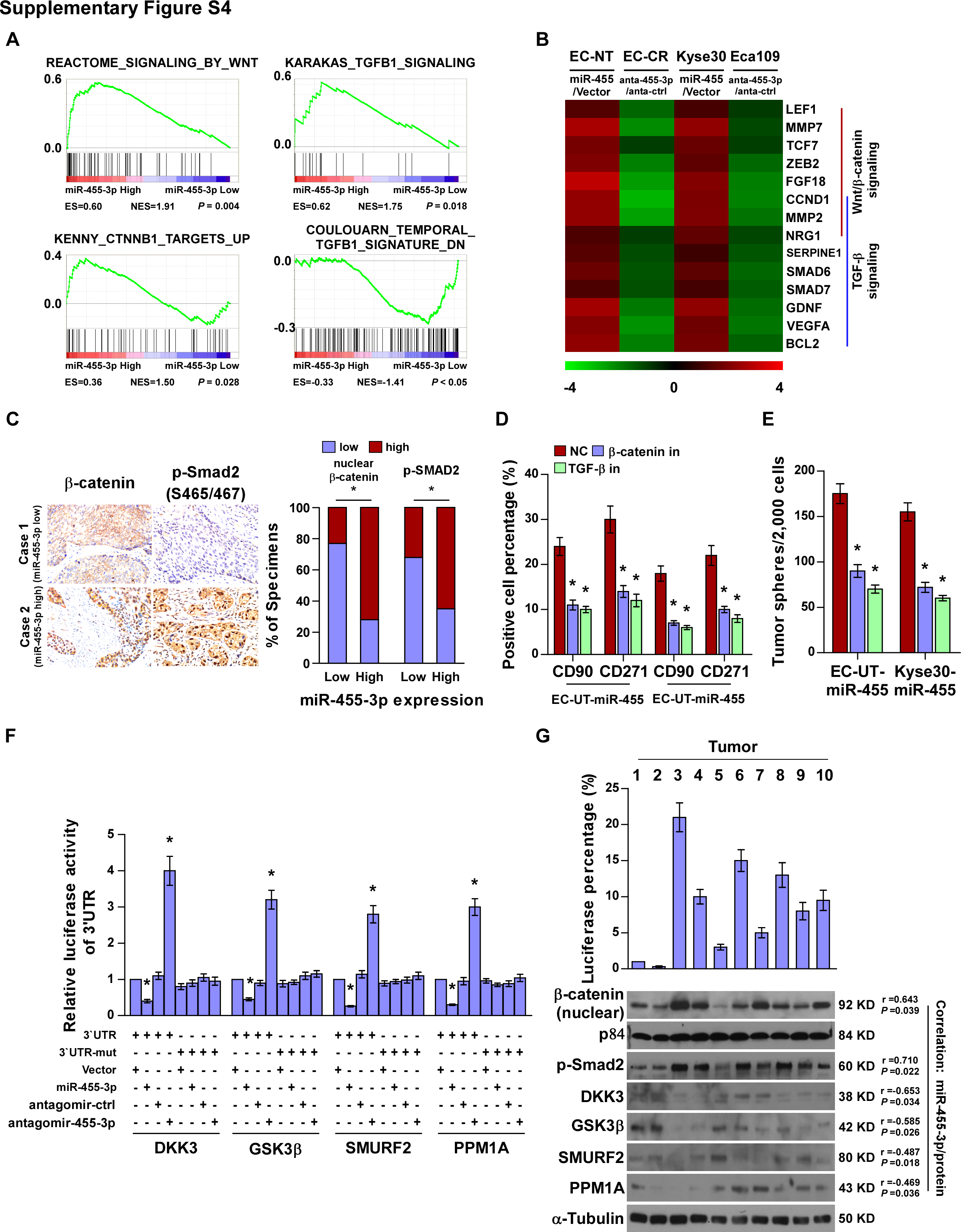

Supplement: Supplementary file 5 — miR-455-3p overexpression activates T-IC-associated signaling pathways. (A) GSEA analysis of TCGA datasets indicating that miR-455-3p expression was significantly correlated with the gene signatures regulated by the Wnt/β-catenin and TGF-β/Smad pathways. (B) Heat map showing real-time PCR results of the downstream target genes of either Wnt/β-catenin or TGF-β signaling in the indicated cells, as compared with corresponding control cells. Pseudo- color scale values were Log2 transformed. (C) miR-455-3p levels were positively correlated with the expression of nuclear β-catenin and p-Smad2 (Ser465/467) in 207 primary human ESCC specimens. Left: Two representative cases are shown. Scale bar: 50 μm. Right: The percentages of specimens showing low or high miR-455-3p expression relative to levels of nuclear β-catenin and p-Smad2 (Ser465/467). (D, E) Quantification of CD90+/CD271+ subpopulations (D) and number of tumorspheres (E) in the indicated cells treated with a β-catenin inhibitor or TGF-β inhibitor. (F) Luciferase assay of the indicated cells transfected with the pGL3-DKK3 (−GSK3β, −Smurf2, −PPM1A) reporter with miR-455-3p mimic, miR-455-3p antagomir or miR-455-3p-mut mimic. (G) Correlation analysis of miR-455-3p with nuclear β-catenin, p-Smad2 (Ser465/467), DKK3, GSK3β, Smurf2, and PPM1A in 10 freshly collected human ESCC samples. Each bar represents the mean ± SD of three independent experiments. *P < 0.05. (TIFF 1465 kb) [file 12943_2017_669_MOESM5_ESM.tif]

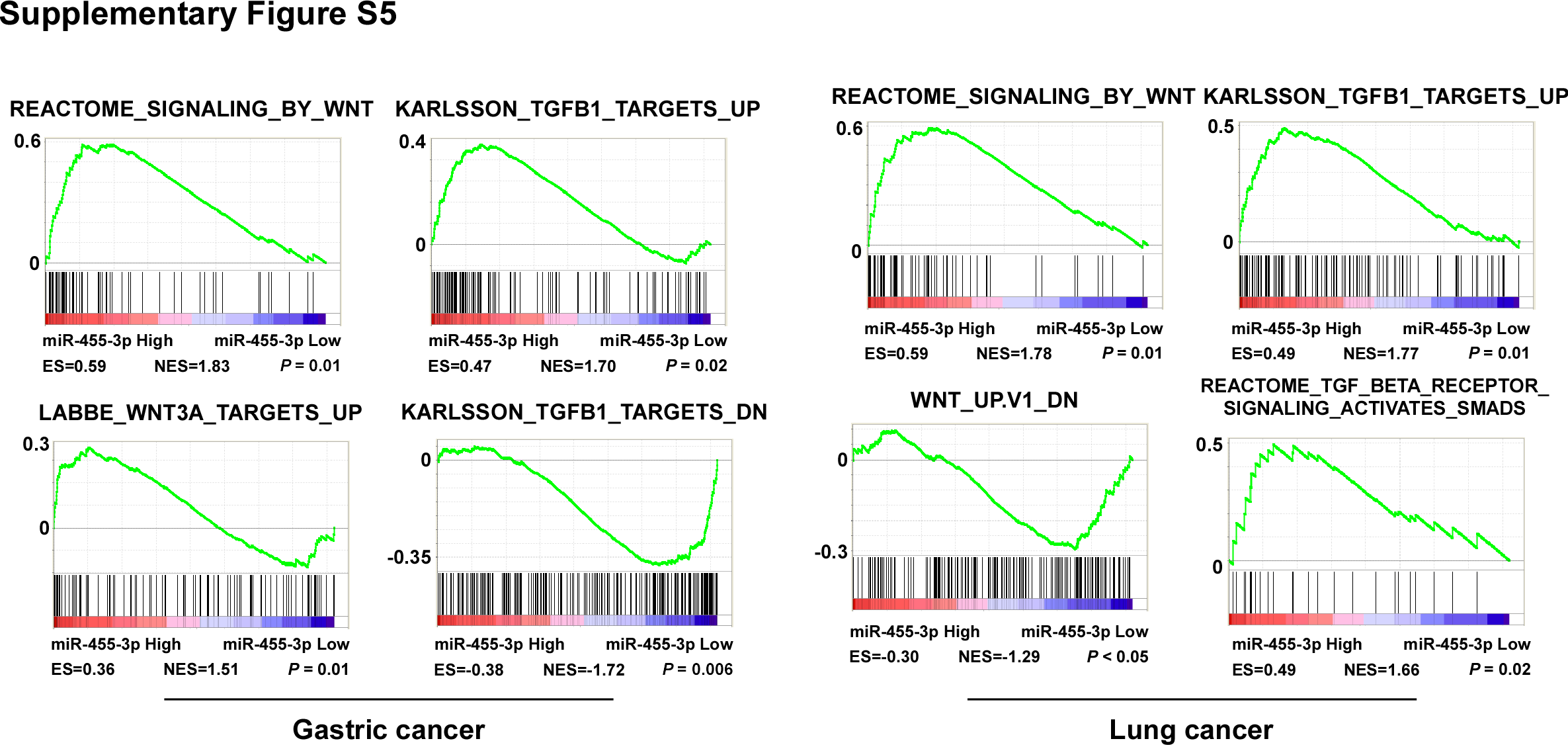

Supplement: Supplementary file 6 — GSEA analysis of TCGA datasets indicating that miR-455-3p levels are correlated with the gene signatures of the Wnt/β-catenin and TGF-β/Smad pathways in gastric and lung cancers. (TIFF 258 kb) [file 12943_2017_669_MOESM6_ESM.tif]
