# Supplementary figures and images for: Correction to: Antagonizing miR-455-3p inhibits chemoresistance and aggressiveness in esophageal squamous cell carcinoma
Source: Mol Cancer. 2021 Dec 1;20:152. doi: 10.1186/s12943-021-01425-4 (PMC8638476; doi:10.1186/s12943-021-01425-4)

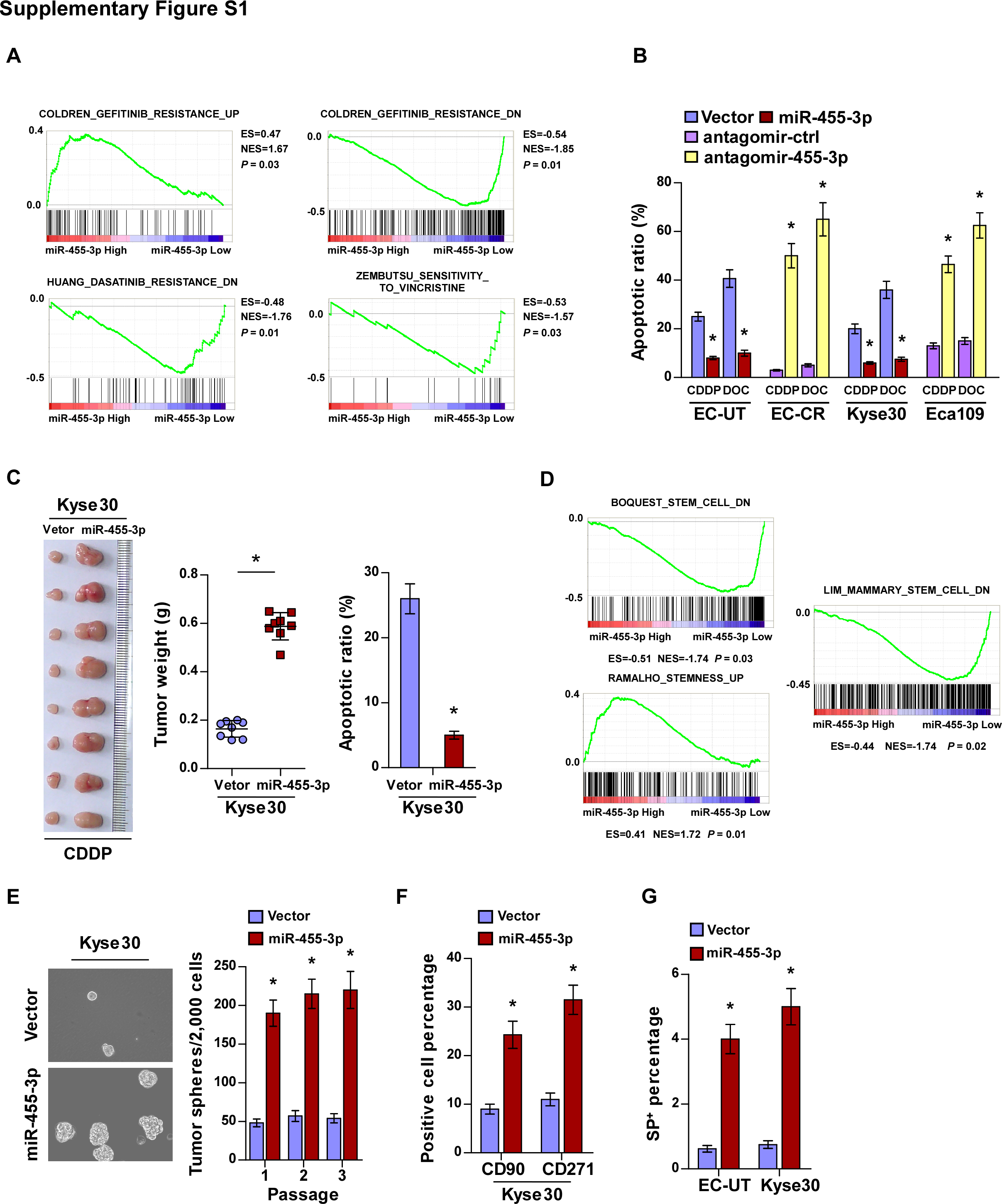

Supplement: Supplementary file 1 — Additional file 1: Supplementary Figure S1. [file 12943_2021_1425_MOESM1_ESM.tif]
